# A Smart Access to the Dinitramide Anion – The Use of Dinitraminic Acid for the Preparation of Nitrogen‐Rich Energetic Copper(II) Complexes

**DOI:** 10.1002/chem.202100747

**Published:** 2021-06-10

**Authors:** Michael S. Gruhne, Maximilian H. H. Wurzenberger, Marcus Lommel, Jörg Stierstorfer

**Affiliations:** ^1^ Energetic Materials Research Department of Chemistry University of Munich (LMU) Butenandtstr. 5–13 81377 München Germany

**Keywords:** burn rate catalyst, dinitramide, dinitraminic acid, laser ignition, primary explosives

## Abstract

Dinitraminic acid (HN(NO_2_)_2_, HDN) was prepared by ion exchange chromatography and acid‐base reaction with basic copper(II) carbonate allowed the *in* *situ* preparation of copper(II) dinitramide, which was reacted with twelve nitrogen‐rich ligands, for example, 4‐amino‐1,2,4‐triazole, 1‐methyl‐5*H*‐tetrazole, di(5*H*‐tetrazolyl)‐methane/‐ethane/‐propane/‐butane. Nine of the complexes were investigated by low‐temperature X‐ray diffraction. In addition, all compounds were investigated by infrared spectroscopy (IR), differential thermal analysis (DTA), elemental analysis (EA) and thermogravimetric analysis (TGA) for selected compounds. Furthermore, investigations of the materials were carried out regarding their sensitivity toward impact (IS), friction (FS), ball drop impact (BDIS) and electrostatic discharge (ESD). In addition, hot plate and hot needle tests were performed. Complex [Cu(AMT)_4_(H_2_O)](DN)_2_, based on 1‐amino‐5‐methyltetrazole (AMT), is most outstanding for its detonative behavior and thus also capable of initiating PETN in classical initiation experiments. Laser ignition experiments at a wavelength of 915 nm were performed for all substances and solid‐state UV‐Vis spectra were recorded to apprehend the ignition mechanism.

## Graphical abstract

**The dinitramide anion** was made accessible for complexation through the use of dinitraminic acid and basic copper(II) carbonate. The *in* *situ* prepared copper dinitramide was complexed by 13 different highly endothermic tri‐ and tetrazole ligands. By the choice of ligand, complexes were obtained that possess thermal stability exceeding 200 degrees Celsius, one even capable of initiating pentaerythritol tetranitrate (PETN). The examined compounds were successfully ignited in laser experiments, with one complex even detonating.

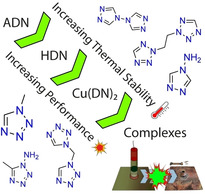

## Introduction

The so‐called ammonium perchlorate composite propellants (APCP) are ammonium perchlorate (AP, NH_4_ClO_4_) (mostly mixed with HTPB and aluminum) based fuels in solid rocket motors.^[1]^ At temperatures above 200 °C, AP decomposes mostly into gaseous chlorine, oxygen, nitrogen, and water due to the reducing effect of the ammonia cation, while the perchlorate anion acts as an oxidizing species.^[2]^ Despite the toxicity of the perchlorate anion and its decomposition products, these mixtures are still widely used, mainly because of their cheap and easy preparation. Nevertheless, much research is being carried out on halogen‐free rocket propellants and burn rate catalysts,^[3]^ with some approaches to replacing AP already well advanced.^[1]^

Besides liquid fuel mixtures based on cryogenic or hypergolic reactions, ammonium dinitramide (ADN) is considered as a compound with high potential.^[1]^ It was first synthesized in 1971 at the Zelinsky Institute of Organic Chemistry in Moscow.^[4]^ However, information was kept under seal and published later in the course of time in the form of some publications.[^4^, ^5^] Therefore, the anion was discovered by US scientists in the late 1980s, independently of the Russians.^[6]^ Since the salt contains no carbon or halogens, its decomposition products are mostly environmentally friendly.^[7]^ This makes the dinitramide anion also a promising candidate for the use in halogen‐free energetic coordination compounds (ECC). These materials could act not only as sensitive explosives but also as low carbon and oxygen‐rich burn rate catalysts, for example, in airbags. Implementing the dinitramide function in 3d transition metal coordination compounds is not a new concept, for example Klapötke *et* *al*. published some dinitramide complexes based on 3‐amino‐1‐nitroguanidines.^[5b,8]]^

**Figure 1:**
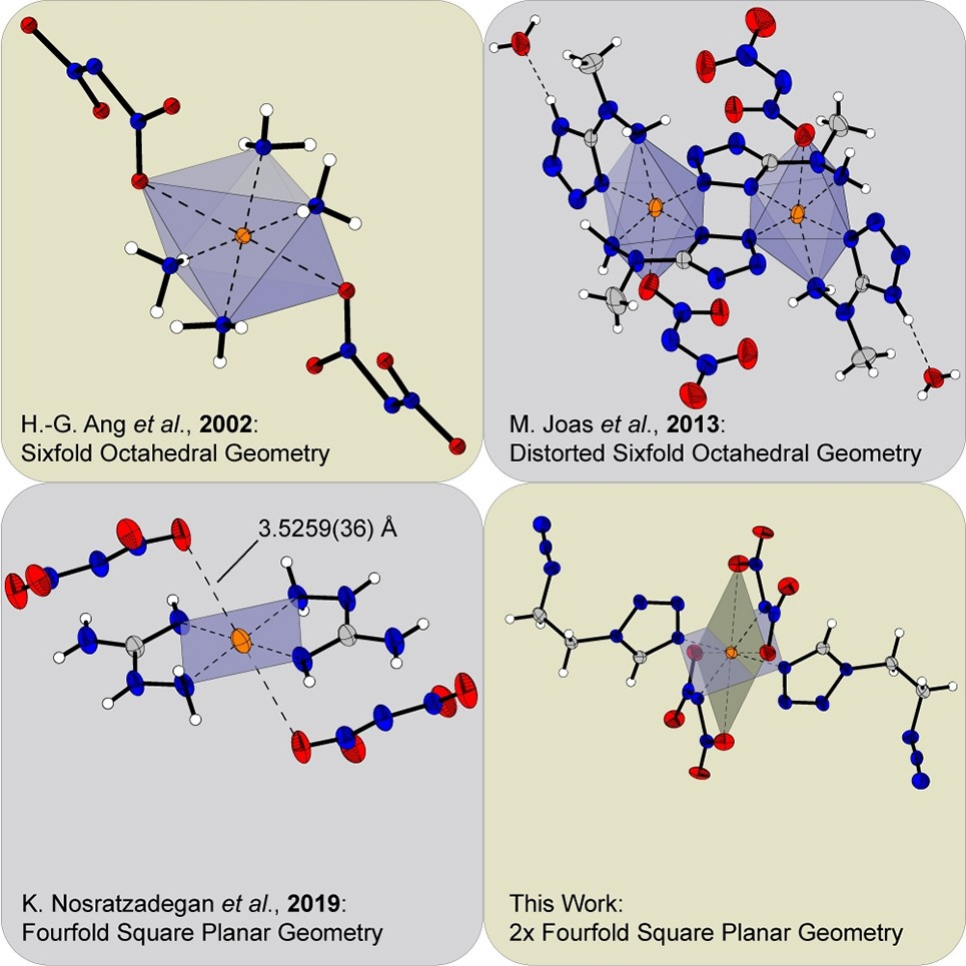
Overview of literature known copper(II) dinitramide ECC and their coordination environments compared to coordination compound **6** of this work.

To the best of our knowledge, only three azole‐based transition metal complexes are known, two of which have been published by the Klapötke group.^[9]^ Both show octahedral coordination geometries, although the one based on 5‐(1‐methylhydrazinyl)‐1*H*‐tetrazole is more clearly distorted (Figure 1). Furthermore, an aminoguanidine‐based complex is known, which exhibits a square planar coordination geometry rare for copper(II) compounds.^[10]^

The low thermal stability of dinitramine‐based compounds (e. g., ADN=135 °C) is a known issue and a significant aspect that must be taken into account when designing new energetic materials based on this anion.^[11]^ At this point, the concept of ECC is applied, since the adaptation of the properties, such as performance and thermal stability, of the formed coordination compounds can be readily tuned by changing the central metal, anion, or ligand.[^12^, ^13^] Szimhardt *et* *al*. already showed that the implementation of propylene bridged ditetrazoles in complexes can increase thermal stability through the linking of the ligand between several metal centers.^[9b]^ Furthermore, a comparison of the previously known complexes shows that probably none of them is able to successfully initiate PETN. Therefore, the focus should also lie on the incorporation of ligands that are known to enhance the performance of the complexes by their high positive heat of formation.[^13b^, ^14^]

The major task that needs to be dealt with when synthesizing dinitramine complexes can be explained by the corresponding tetraammine copper(II) compound (**2**) presented in Figure 1. The coordination compound was already described in 2002 and, due to the impossible isolation of an elemental pure sample, was only examined by X‐ray diffraction, ^14^N NMR, and vibrational spectroscopy.^[15]^ The reason for this is probably the reaction that precedes the formation of the complex. Typically, a mixture of ammonium dinitramide with any copper(II) salt is reacted with the respective ligand.^[9b]^ Alternatively, a copper(II) complex with any anion and the desired ligand is formed in solution and a dinitramide salt is added.^[8]^ Thus, in every case, ions are left behind which can contaminate the sample or lead to a wrong composition of the complex. In this work the problem was solved using ion exchangers, which enables the simple production of dinitraminic acid (HDN). Reaction with metal carbonates allows the preparation of pure metal dinitramide salts without interfering anions *in* *situ*, which was already shown by Lukyanov *et* *al*. by reacting HDN with silver carbonate.^[5b]^ However, the dinitraminic acid was preferably obtained by the injection of hydrogen chloride into a solution of KDN. The idea of an alternative preparation of an aqueous HDN solution was already shown by Bottaro *et* *al*. though the authors did not perform reactions with metal carbonates of the 3d series.^[6]^

The complexes synthesized in this way can be used in a wide range of applications. The easily accessible anion is not only extending the field of ECC with this method, but the resulting complexes can also serve as burn rate catalysts in solid propellants and as halogen‐ and lead‐free explosives. In addition to this, the complexes can also be used in the field of laser‐ignitable compounds.^[16]^

## Results and Discussion

### Synthesis

**Scheme 1:**
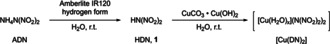
Preparation of an aqueous solution of copper(II) dinitramide.

As already mentioned above, the synthesis of dinitramide‐based coordination compounds in particular has some drastic disadvantages. Until now, in every case ammonium dinitramide (ADN) or potassium dinitramide (KDN) was used as a starting material together with water‐soluble copper(II) salts.[^8^, ^9^, ^10^, ^15^] This leads to the possible formation of complexes without the integration of dinitramide anions. In the case of chloride anions, which is known to act as a bridge between metal centers, the formation of such complexes may even be facilitated.^[17]^ To avoid such side reactions, ion exchange techniques were used. Ammonium dinitramide was thus converted into a highly clean aqueous solution of dinitraminic acid. The solution obtained should be practically free of interfering anions and can be applied for the *in* *situ* formation of transition metal dinitramides when reacted with the corresponding carbonates (Scheme 1).

Due to the instability of pure dinitraminic acid at ambient conditions special attention is required during the preparation of the acid and an isolation of the pure compound has to be avoided.^[18]^ Concentrations in the range of 7–10 wt % can be safely prepared in aqueous solution. At a concentration of about 20 wt % the solution is still stable for several days. Higher concentrations should be avoided as decomposition can occur explosively.^[5b]^ In addition to photocatalyzed decomposition of the dinitramide anion in aqueous solution, low stability in acids must be taken into account.[^18^, ^19^] It is reported that the decomposition of HDN acid is catalyzed in 8 m sulfuric acid. In 10 m sulfuric acid the decomposition takes place within minutes.^[18]^ This could be an important fact as ion exchange materials can be regenerated by acids.

**Scheme 2:**
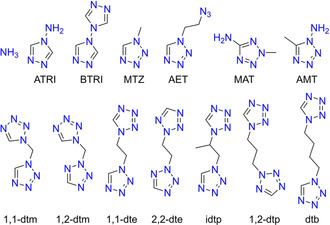
Ligands used for the complexation of copper(II) dinitramide.

In order to provide a general proof of concept, the ammine ligand was chosen as the resulting complex could not be obtained in a clean manner by the common methodology (Scheme 2).^[15]^ The use of nitrogen‐rich endothermic tri‐ and tetrazole ligands increases the energetic character of the ECC and, therefore, several different mono‐ and ditetrazoles were selected.[^13^, ^14^, ^20^] Since dinitramide‐based compounds are known to suffer from low thermal stability,^[11]^ the main focus was on bridging molecules, such as 1,2,4‐triazoles and ditetrazoles.^[12]^ Since the further aim of this work was to increase the energetic character of the complexes, focus was also put on ligands like 1‐AT, 1‐AMT, 1,5‐DAT, as well as with different dtm isomers (Scheme 2). These ligands possess the highest calculated gas phase enthalpies of formation and are therefore known to yield the most powerful coordination compounds.[^13^, ^14^]

**Scheme 3:**
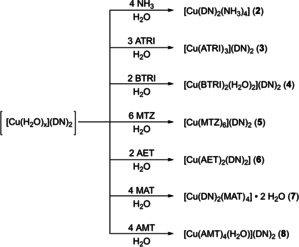
Overview of the prepared ECC **2**–**8**.

**Scheme 4:**
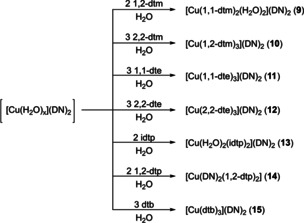
Overview of the prepared ditetrazole based copper(II) dinitramide coordination compounds **9**–**15**.

All used ligands (Scheme 2) were either purchasable (NH_3_, ATRI) or synthesized using literature known procedures. Tetrazole derivatives were either prepared by the reaction of alkyl halides (MAT, dtm, 2,2‐dte, 1,2‐dtp) or dimethyl sulfate (1‐MTZ) with 1,5*H*‐tetrazole[^9b^, ^13a^, ^14^, ^20a^, ^21^] and through ring closure reactions using sodium azide, triethyl orthoformate, and the respective alkyl amine or diamine (AET, AMT, 1,1‐dte, idtp, dtb).[^13b^, ^22^] For the syntheses of the complexes, freshly prepared aqueous solutions of copper(II) dinitramide as well as stochiometric amounts of the respective ligands were combined (Scheme 3 & Scheme 4) and the resulting reaction mixtures were left for crystallization at ambient conditions.

All coordination compounds were directly obtained from the mother liquors and crystallized in moderate to excellent yields (55–92 %). Single crystals, suitable for X‐ray diffraction were directly obtained except for **3**, **4**, and **14**. Regarding compounds **3** and **14**, the complex’ composition was determined by elemental analysis. Sufficient single crystals of ECC **4** were obtained through layering experiments.

The crystalline materials were filtered off, washed with a small amount of ice‐cold water when necessary and dried in air overnight. The use of other common monotetrazoles such as 1‐amino‐5*H*‐tetrazole,^[17]^ 1,5‐dimethyltetrazole,^[23]^ 1,5‐diaminotetrazole,^[24]^ and 1‐methyl‐5‐aminotetrazole^[25]^ or ditetrazoles such as di(tetrazol‐2‐yl)propane,^[9b]^ which have already been successfully used as ligands, did not result in isolable crystalline ECC. One factor that could play a crucial role here is that in our synthesis we were limited to the solvent water because of the ion exchange and could not use organic solvents such as acetonitrile.

### Crystal structures

All compounds, with the exception of complex **2**, whose crystal structure is already known in the literature, and compounds **3** and **14**, where it was impossible to obtain crystals of sufficient quality, were characterized by low temperature X‐ray diffraction. In case of compounds **5** and **7** the measurements allow an indication of the most likely composition, but finalization of the data sets was not possible due to the highly disordered dinitramide moieties. The complexes’ composition was finally confirmed by elemental analysis. Copper atoms are displayed as orange ellipsoids, nitrogen atoms blue, oxygen atoms red, carbon atoms grey and hydrogen atoms white. Further information on the structures of the compounds **8**, **10**, and **11** together with the measurement and refinement data of all crystallograpically investigated compounds are given in the Supporting Information (Tables S1–3, and Figures S1–3). The crystal datasets were uploaded to the CSD database^[26]^ and can be obtained free of charge. The bond lengths and angles of the coordinating ligands in the crystallographically investigated complexes are within the typical range of tetrazole and triazole ligands and nearly the same as in the non‐coordinating molecules.[^9b^, ^13b^, ^14^, ^21^, ^22^, ^27^] Therefore, they are not part of the discussion in any of the following coordination compounds. All copper(II) dinitramide coordination compounds except ECC **6** and **8** show an octahedral coordination sphere around the central metal with typical Jahn‐Teller distortions along the axial axis.

**Figure 2:**
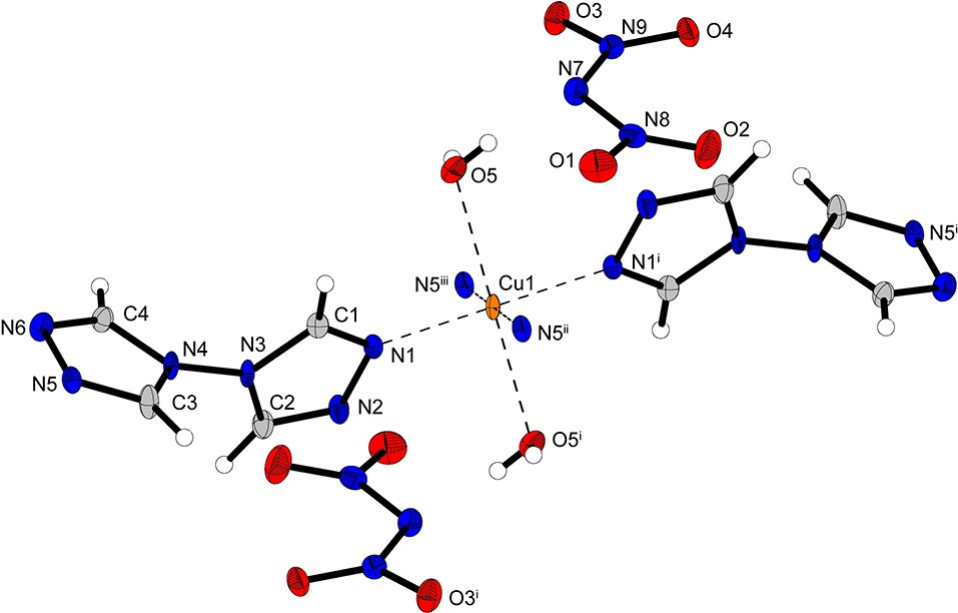
Coordination environment of [Cu(BTRI)_2_(H_2_O)_2_](DN)_2_ (**4**). Selected bond lengths (Å): Cu1−N1 2.003(2), Cu1−N5^ii^ 2.034(2), Cu1−O5 2.356(3); selected bond angles (°): O5−Cu1−N1 89.77(9), O5−Cu1−N5^ii^ 94.57(10), N1−Cu1−N5^ii^ 88.87(10). Symmetry codes: (i) −x, −y, −z; (ii) −0.5+x, −0.5−y, −0.5+z; (iii) 0.5−x, 0.5+y, 0.5−z.

The triazole‐based coordination compound **4** crystallizes in the monoclinic space group *P*2_1_/*n* in the form of blue blocks. The unit cell is built up by two formula units and possesses a calculated density of 1.945 g cm^−3^ at 120 K. The octahedral coordination sphere consists of two BTRI in equatorial and two aqua ligands in axial position (Figure 2). The triazoles are linking between two different copper centers, building up 2D polymeric layers.

**Figure 3:**
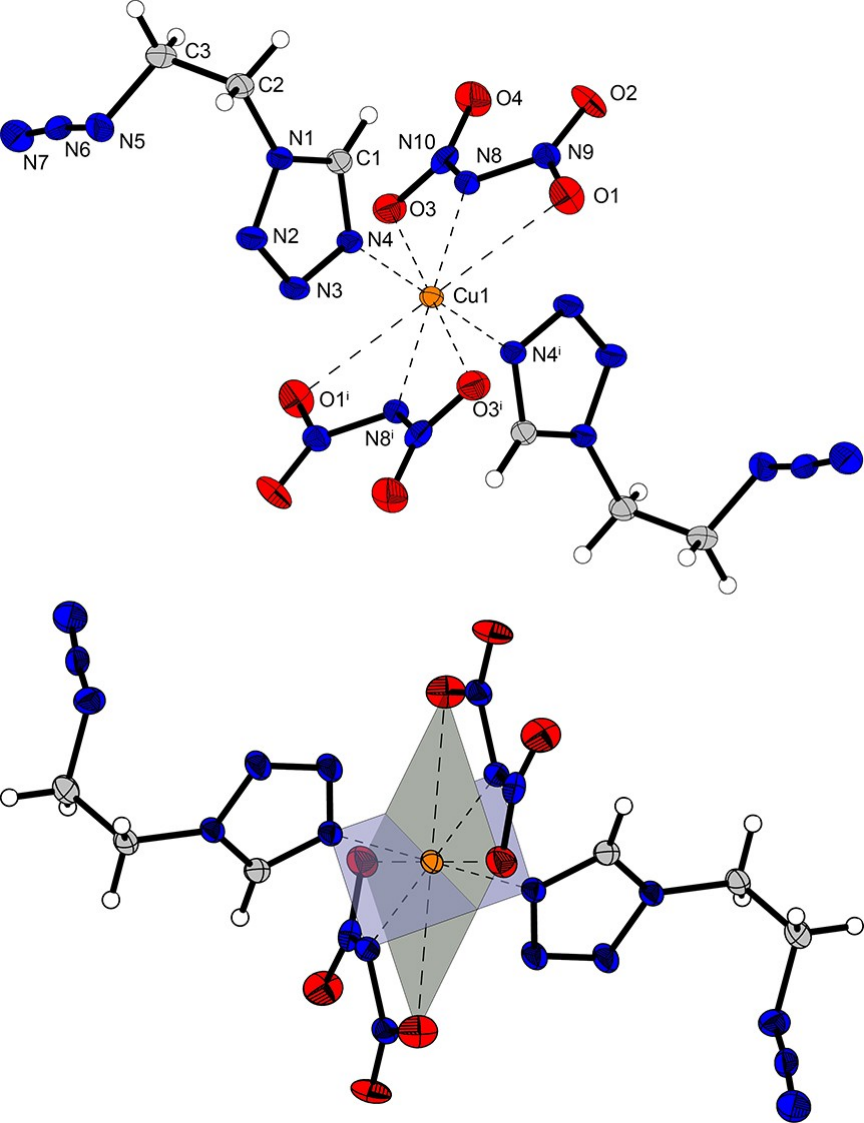
Molecular unit of [Cu(AET)_2_(DN)_2_] (**6**) (top) and illustration of the double square planar coordination sphere around the central metal (bottom). Selected bond lengths (Å): Cu1−N4 1.975(2), Cu1−N8 1.999(2), Cu1−O1 2.782(2); selected bond angles (°): O1−Cu1−O3 98.23(6), O1−Cu1−N4 86.47(7), O3−Cu1−N8 49.22(7), N4−Cu1−N8 90.76(8). Symmetry code: (i) 1−x, 1−y, 1−z.

Dinitramide complex **6** shows a very uncommon coordination geometry around the copper(II) central metal and crystallizes in the monoclinic space group *P*2_1_/*c* in the form of blue needles. The unit cell is built up by two formula units and possesses a calculated density of 1.932 g cm^−3^ at 102 K. The complex monomers are formed by two neutral AET molecules and two dinitramide anions bounded to the metal cation (Figure 3). The tetrazole ligands and the deprotonated amide moieties form a square planar coordination sphere with bond lengths below 2 Å. The very unusual fourfold coordination around the Cu^2+^ cation is stabilized by another square planar plane formed by four oxygens of the nitro groups with very weak Cu−O interactions in the range of ∼2.7–2.8 Å. Both coordination tiers are perfectly planar (N4−N4^i^−N8−N8^i^=0°, O1−O1^i^−O3−O3^i^=0°) and almost perpendicular to each other. This results in an eightfold coordination sphere, which is to be expected for metals of the 4d series but is very rare within the 3d metals. This is most likely made possible by the additional square planar coordination by the oxygen atoms of the dinitramido ligands mentioned earlier. The Cu1−O3, bond is therefore with a length of 2.858(2) Å rather long compared to the other bonds. It is difficult to determine exactly which complexes are present in solution, but it is likely that the complex formed here is the most thermodynamically stable in the solid state.

**Figure 4:**
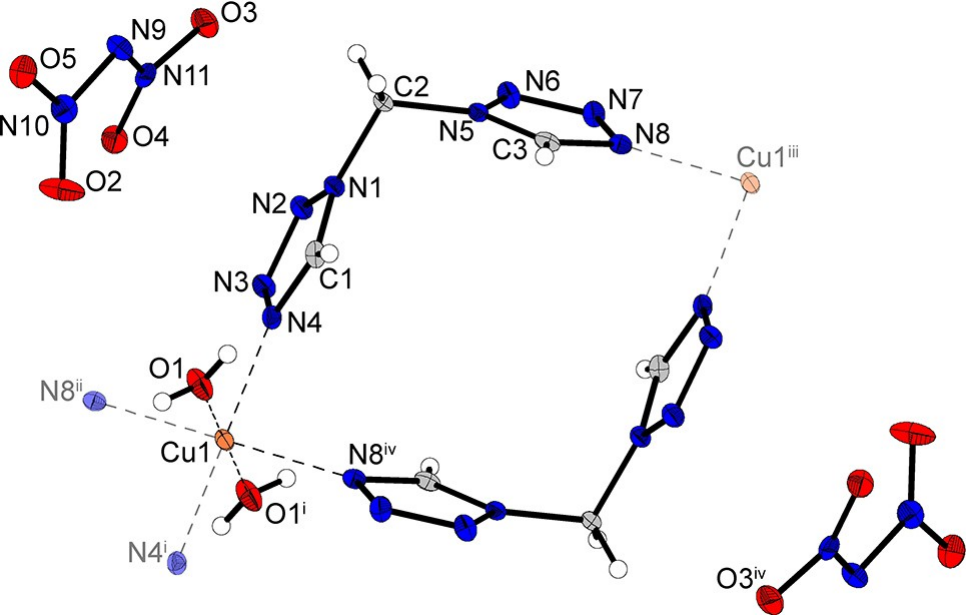
Extended coordination environment of [Cu(1,1‐dtm)_2_(H_2_O)_2_](DN)_2_ (**9**). Selected bond lengths (Å): Cu1−N4 2.0289(19), Cu1−O1 2.2484(19) Cu1−N8 2.051(2); selected bond angles (°): N4−Cu1−N8 89.54(8), N4−Cu1−O1 89.32(8), N8−Cu1−O1 92.56(8). Symmetry codes: (i) −x, 2−y, 1−z; (ii) −1+x, y, z; (iii) 1+x, y, z; (iv) 1−x, 2−y, 1−z.

Energetic coordination compound **9** crystallizes in the monoclinic space group *P*2_1_/*n* in the form of blue blocks. The unit cell is built up by two formula units and possesses a calculated density of 2.017 g cm^−3^ at a temperature of 123 K. The coordination sphere is arranged similarly to complex **4**, consisting of two bridging ligand units in equatorial positions and two aqua ligands in axial position (Figure 4). Despite this correlation, the polymeric structure of both complexes is different due to the different binding behavior of the triazole‐based ligand (**4**) compared to the tetrazole derivative in ECC **9**. Since two 1,1‐dtm ligands each bind to the same metal center, only chains are formed instead of 2D layers. The occupation of the axial positions by the water molecules is most likely made possible by the steric hindrance of the ligands caused by the methylene bridge, which prevents coordination of further ligand moieties. Nevertheless, anhydrous copper(II) complexes based on 1,1‐dtm have recently been discovered by our research group.^[14]^ This requires the use of organic solvents, such as acetonitrile, to prevent the coordination of aqua ligands but is impossible in this case due to the use of aqueous HDN solution.

**Figure 5:**
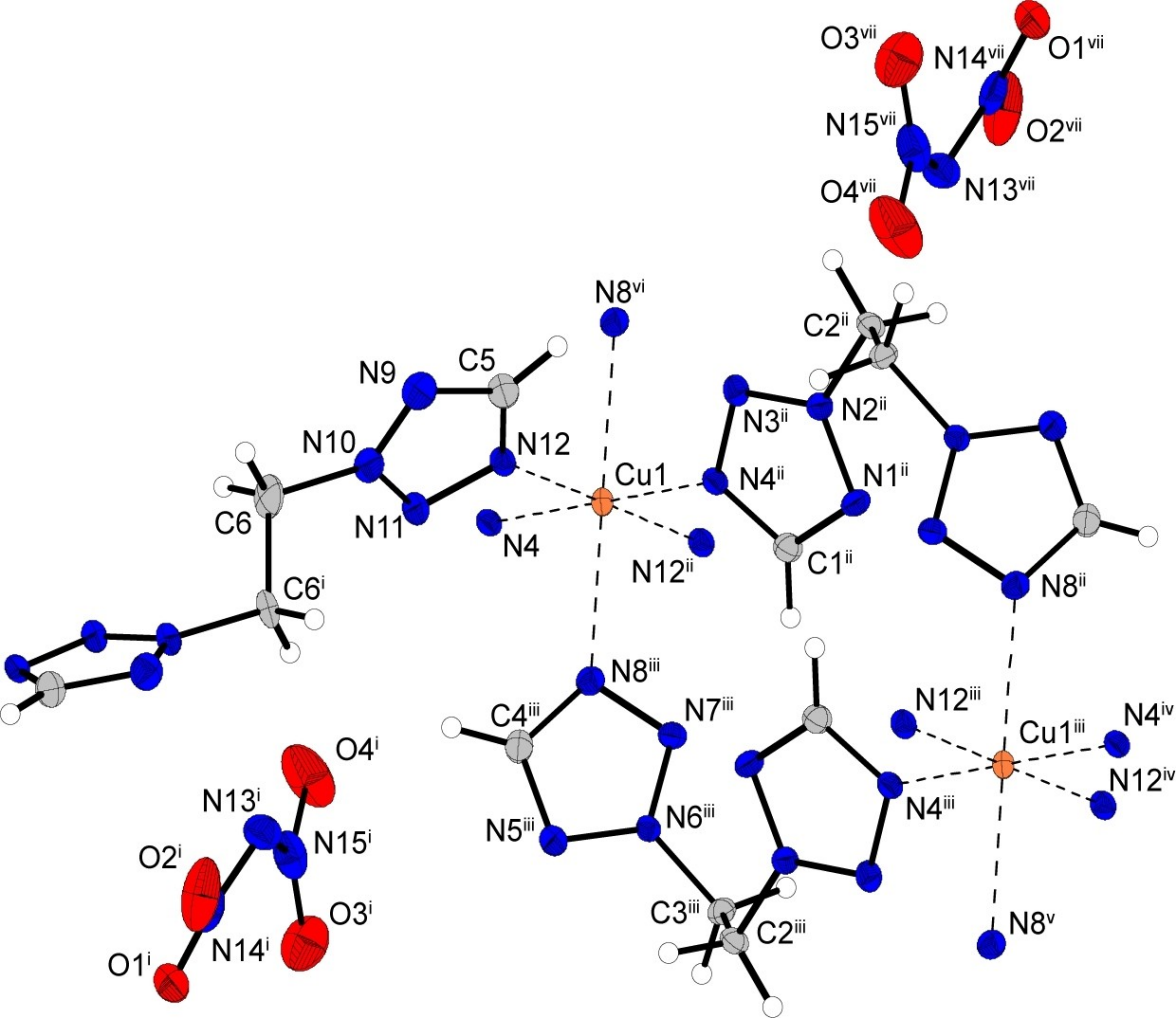
Extended coordination environment of [Cu(2,2‐dte)_3_](DN)_2_ (**12**). Selected bond lengths (Å): Cu1−N4 2.0320(18), Cu1−N8 2.3833(18), Cu1−N12 2.0260(17); selected bond angles (°): N12−Cu1−N8 88.42(7), N4−Cu1−N12 88.88(7), N4−Cu1−N8 88.99(7), selected torsion angles (°): N2−C2−C3−N6 71.87(18), N10−C6−C6^i^−N10^i^ 178.146(2). Symmetry codes: (i) 1−x, y, 1.5−z; (ii) 0.5−x, 0.5−y, 1−z; (iii) −0.5+x, −0.5+y, z; (iv) −x, −y, 1−z; (v) −1+x, −1+y, z; (vi) 1−x, 1−y, 1−z; (vii) −0.5+x, 0.5−y, −0.5+z.

The copper(II) dinitramide complex **12** crystallizes as blue blocks in the monoclinic space group *C*2/*m* with one formula unit per unit cell and a calculated density of 1.815 g cm^−3^ at 123 K. The coordination sphere is built up analogous to compound **11**. Three (2,2‐ditetrazolyl)ethane ligands, each linking between two copper centers are part of the molecular unit (Figure 5). The Jahn‐Teller distortion is located along the N8−Cu1−N8 axes.

**Figure 6:**
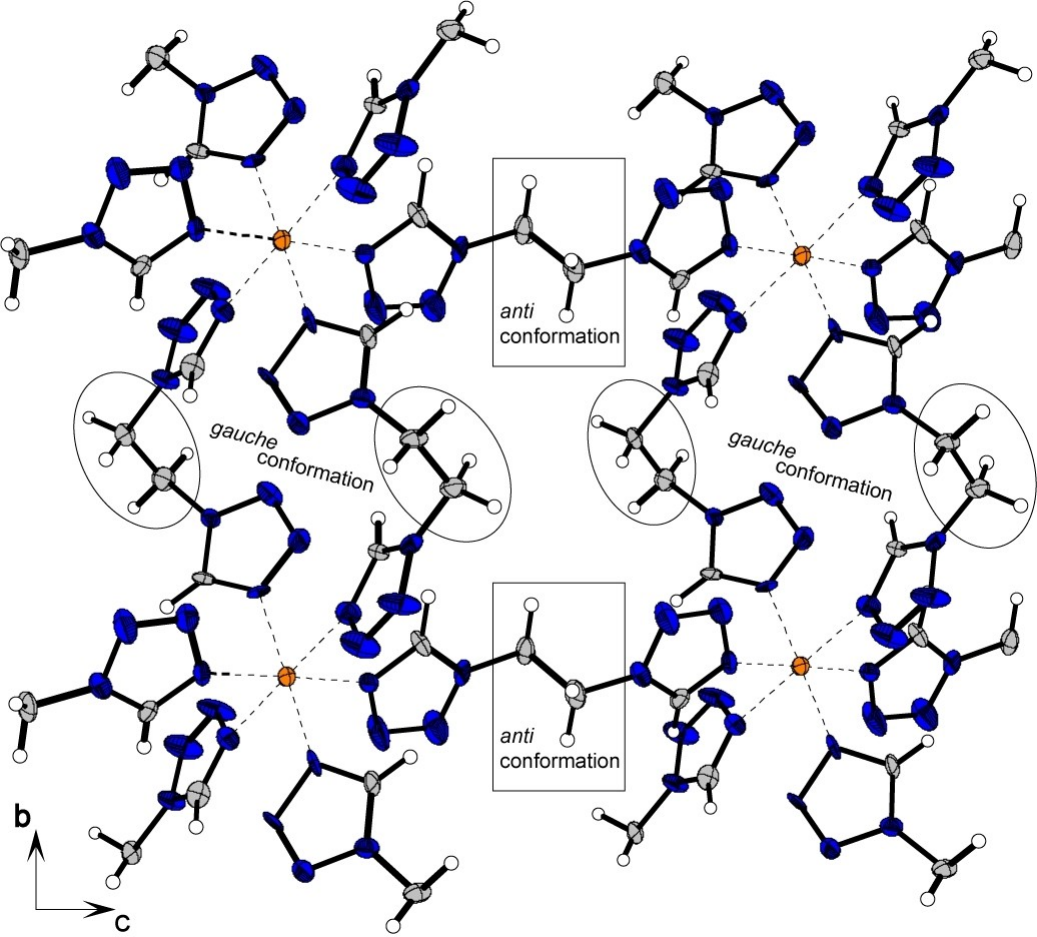
Two‐dimensional layers of compound **11** along the *a* axis.

**Figure 7:**
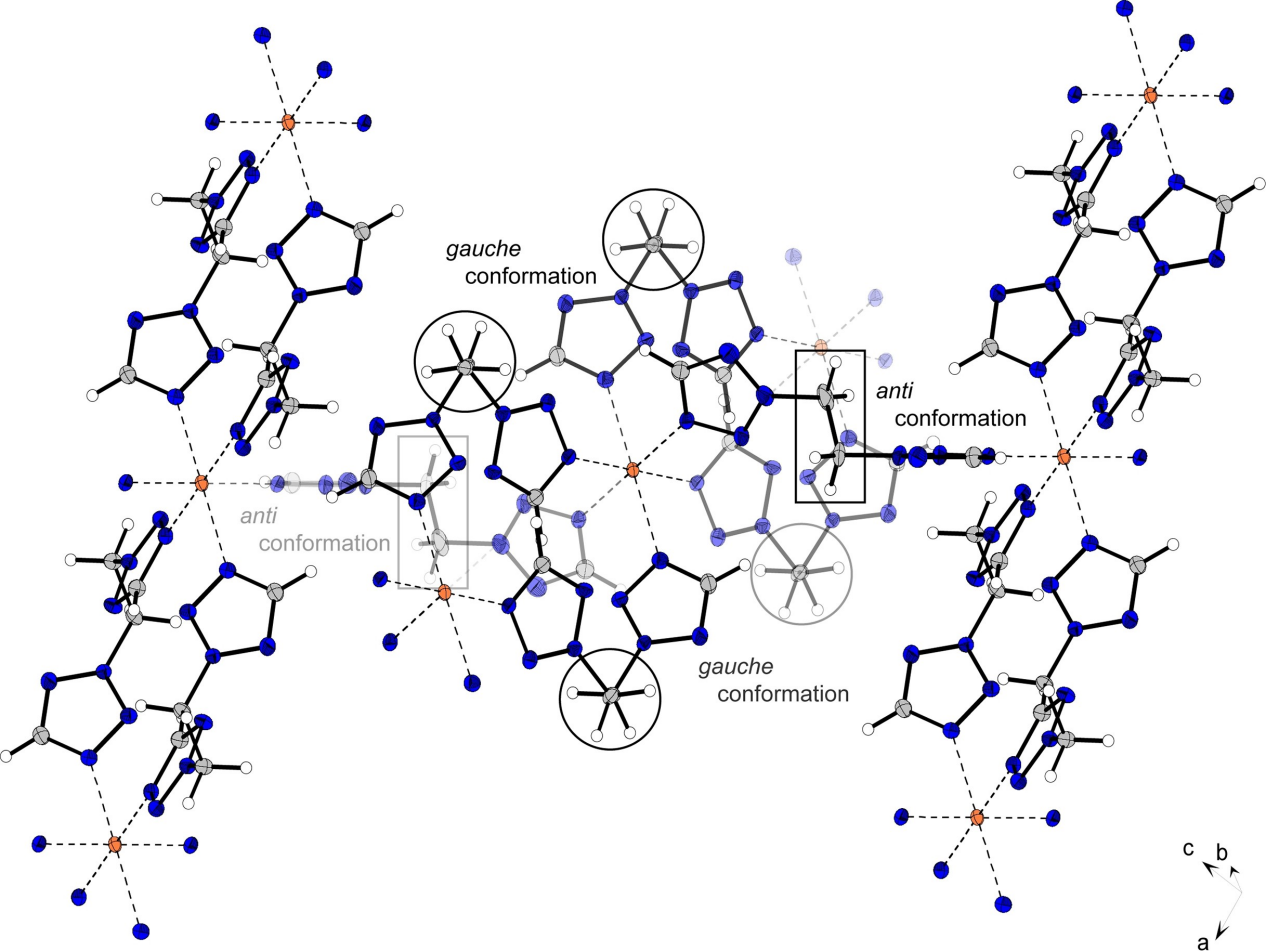
Polymeric chains formed by ligands in *gauche*‐conformation and linked to form a 3D network by ligands showing an *anti*‐arrangement in compound **12**.

Two different bonding types of the ditetrazole ligand can be observed in the two similar crystallizing complexes **11** and **12**. Each coordination compound possesses two ligands in *anti*‐conformation, together with one ligand arranged linearly in *gauche* conformation (Figures 6 and 7). In every case investigated, the Jahn‐Teller distortion is found between ligands in *anti*‐conformation and the copper center.

The two ligands arranged in *gauche*‐conformation bridge between the same copper centers and thus form 1D polymeric chains (Figure 6). These networks are linked further by the ligands in *anti*‐conformation. However, the type of substitution of the ligands plays a crucial role in the type of organometallic framework formed. The 1,1‐substitution in **11** leads to the formation of two‐dimensional sheets, whereas the 2,2‐substitution in **12** results in the formation of a three‐dimensional structure (Figure 7).

**Figure 8:**
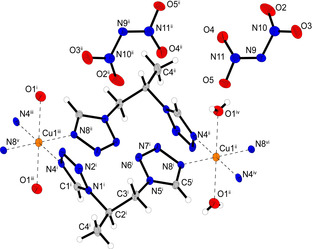
Section of the coordination environment of compound **13**. Selected bond lengths (Å): Cu1^ii^−N4^ii^ 2.02595(12), Cu1^ii^−N8^i^ 2.01064(14), Cu1^ii^−O1^iv^ 2.36080(16); selected bond angles (°): N4^ii^−Cu1^ii^−O1^iv^ 94.223(5), N4^ii^−Cu1^ii^−N8^i^ 89.992(7), N8^i^−Cu1^ii^−O1^iv^ 87.337(5). Symmetry codes: (i) −x, 1−y, −z; (ii) −0.5+x, 1.5−y, −0.5+z; (iii) −1+x, y, z; (iv) 1−x, 1−y, −z; (v) −1.5+x, 1.5−y; (vi) 1.5−x, 0.5+y, 0.5−z.

Coordination compound **13** crystallizes as blue rods in the monoclinic space group *P*2_1_/*n* with two formula units per unit cell. The complex possesses a calculated density of 1.753 g cm^−3^ at 106 K, which is the second lowest density observed among all crystallographically investigated coordination compounds in this work. The coordination sphere is built up similar to the one observed for compound **9**. The *gauche* conformation of the ligand's carbon chains is showing the same effect, as already found in the structures of complexes **10** and **11**. Two ligand moieties are linking between the same copper centers, forming one‐dimensional chains (Figure 8).

**Figure 9:**
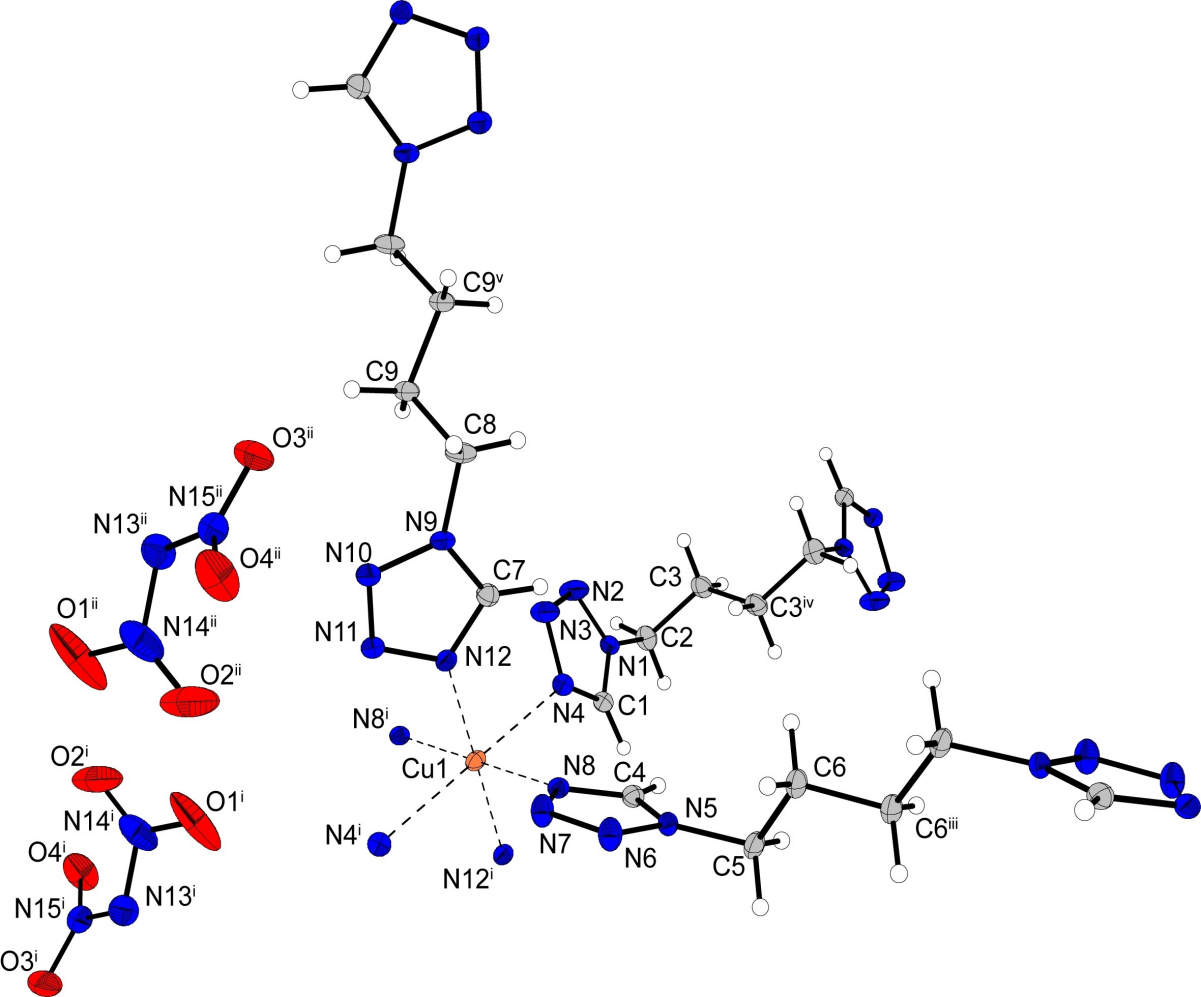
Extended coordination environment of compound **15**. Selected bond lengths (Å): Cu1−N4 2.4811(17), Cu1−N8 1.9967(14), Cu1−N12 2.032(2); selected bond angles (°): N4−Cu1−N12 90.71(7), N4−Cu1−N8 90.18(6), N8−Cu1−N12 89.98(7). Selected torsion angles (°): N1−C2−C3−C3^i^ −60.1(3), C2−C3−C3^i^−C2^i^ 180.0(3), C3−C3^i^−C2^i^−N1^i^ 60.1(3). Symmetry codes: (i) −x, 2−y, 1−z; (ii) 1−x, 1−y, 2−z.

Complex **15** crystallizes in the triclinic space group *P* $\bar{1}$ in the form of blue blocks. The unit cell consists of one formula unit and with a density of 1.663 g cm^−3^ at 108 K it is the least dense of all compounds crystallographically investigated during this study. The octahedral coordination sphere is built up by six di(tetrazolyl)butane units, each bridging to another copper center (Figure 9).

**Figure 10:**
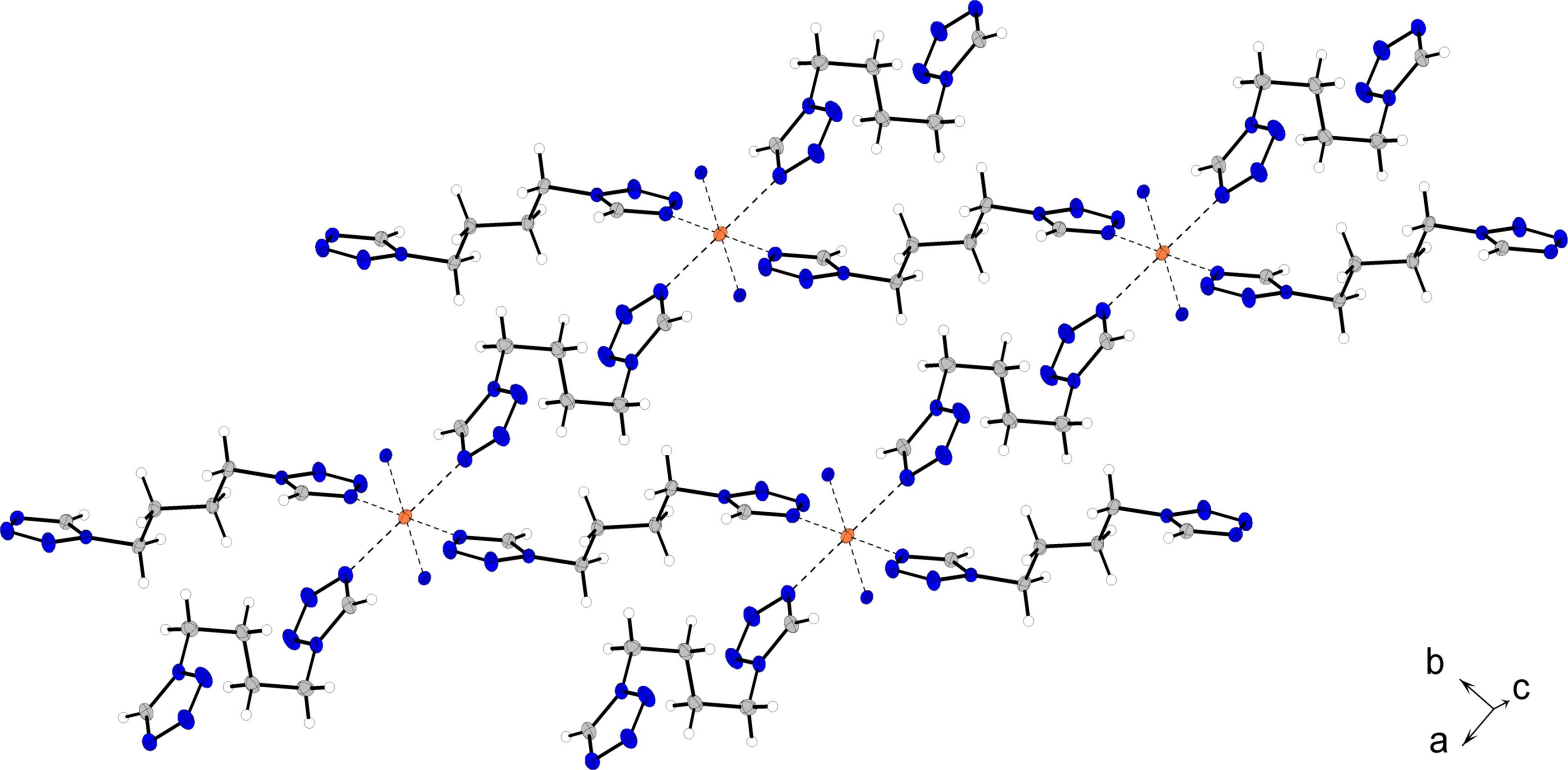
Section of the framework, which is formed in complex **15**. The ligands, not displayed for simplicity, are also aligned in *anti*‐conformation and form 2D layers.

Unlike coordination compounds **11** and **12**, two ligands are arranged in *anti*‐conformation, forming planar 2D polymeric layers. The C3−C3^i^ bond of the remaining ligand also shows an *anti*‐conformation. However, the ligand's remaining carbon bonds (C2−C3, C3^i^−C2^i^) are in a *gauche* pattern. Due to the length of the carbon chain this leads to a linearly aligned ditetrazole, which links between the two‐dimensional layers and builds up a three‐dimensional polymeric network (Figure 10).

### Sensitivities and thermal stability

**Figure 11:**
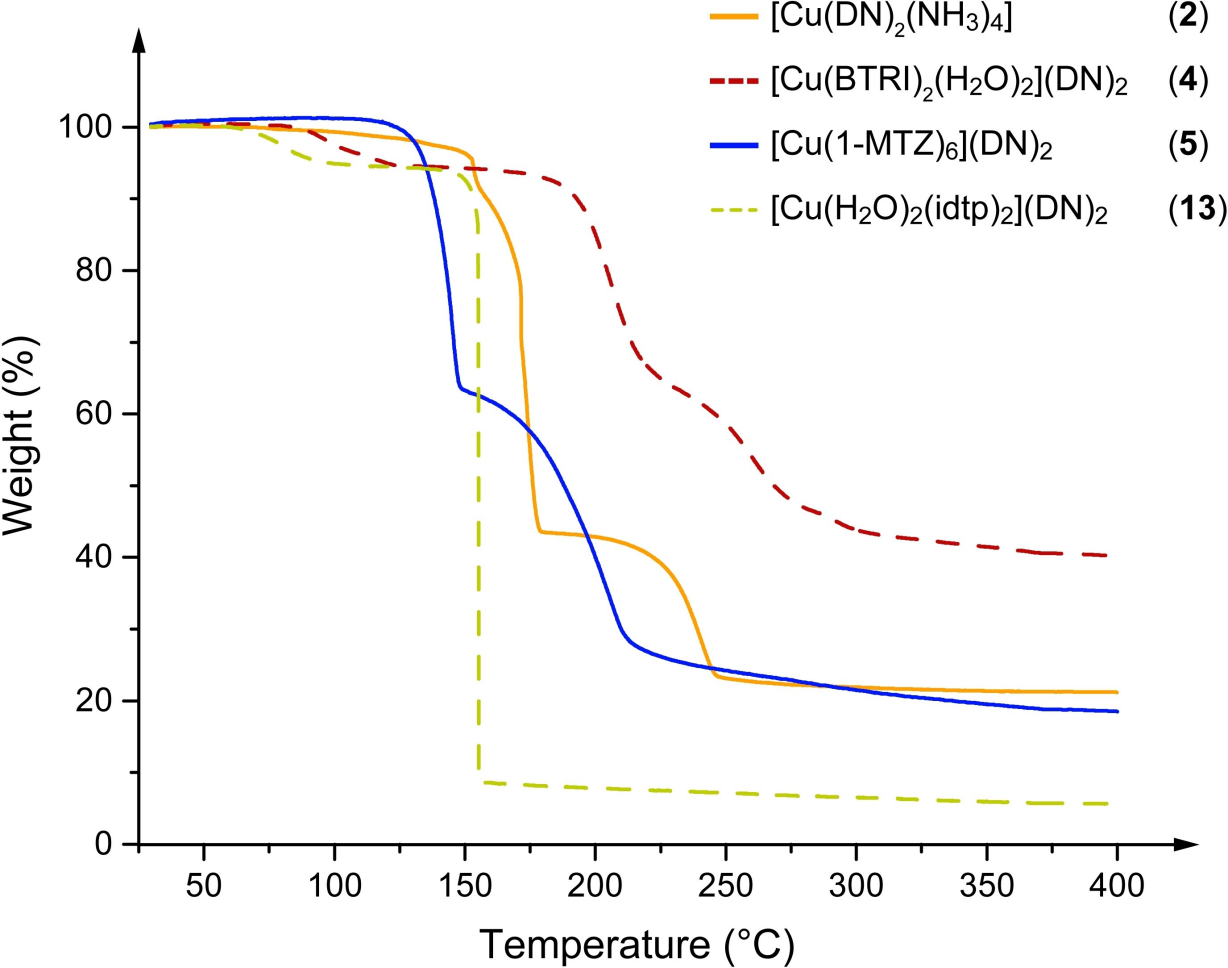
TGA spectra of the coordination compounds **2**, **4**, **5**, and **13** in the range of 30 °C to 400 °C at a heating rate of 5 °C min^−1^.

Every coordination compound was investigated by differential thermal analysis (DTA) at a linear heating rate of 5 °C min^−1^ in the range of 25–400 °C. The observed decomposition temperatures as well as endothermic events (melting or loss of aqua ligands and crystal water) are listed in Table 1. In addition, thermogravimetric (TGA) measurements at the same heating rate and temperature range were carried out for compounds **2**, **4**, **5**, **8**, **9**, **13**, and **15** (Figures 11, S10–S11) in order to investigate the behavior of the complexes on heating in more detail. The DTA plots of compounds **2**–**15** as well as additional information regarding the thermal stabilities can be found in the in the Supporting Information (Figures S7–S9).

**Table 1:** Overview on the compounds’ thermal stability^[a]^ according to DTA.

No.	*T*_endo_ [°C]^[b]^	*T*_exo_ [°C]^[c]^	No.	*T*_endo_ [°C]^[b]^	*T*_exo_ [°C]^[c]^
**2**	66	179	**9**	97	165
**3**	–	203	**10**	–	147
**4**	129	195	**11**	–	164
**5**	114	159	**12**	–	150
**6**	–	106	**13**	113	152
**7**	65	114	**14**	–	141
**8**	89^*^	89^*^	**15**	97	147

[a] Onset temperature at a heating rate of 5 °C min^−1^ measured by DTA; [b] Endothermic peak, which indicates melting or loss of aqua ligands; [c] Exothermic peak, which indicates decomposition; * Endothermic event followed by exothermic event.

Endothermic events were observed for eight of the fourteen investigated compounds. As verified by TGA, the endothermic events of **4**, **9**, and **13** represent the loss of aqua ligands or, in the case of compound **7**, the loss of crystal water. Thermogravimetric measurements of the compounds **2**, **5**, **8**, and **15** indicated no loss of mass before reaching the decomposition temperature of the complexes. This indicates that the endothermic events only represent melting points of the ECC **2**, **5**, and **15**. The DTA measurement of complex **8** has an endothermic event immediately followed by an exothermic event, thus it is assumed that the loss of the aqua ligand (*T*_endo_=89 °C) simultaneously results in the decomposition of the complex. The reason for this is probably a square planar complex resulting from the loss of the ligand. Its stability is so low that decomposition occurs immediately after dehydration. In consequence, no mass loss prior to decomposition could be detected during the TG measurement since both events occur simultaneously.

**Figure 12:**
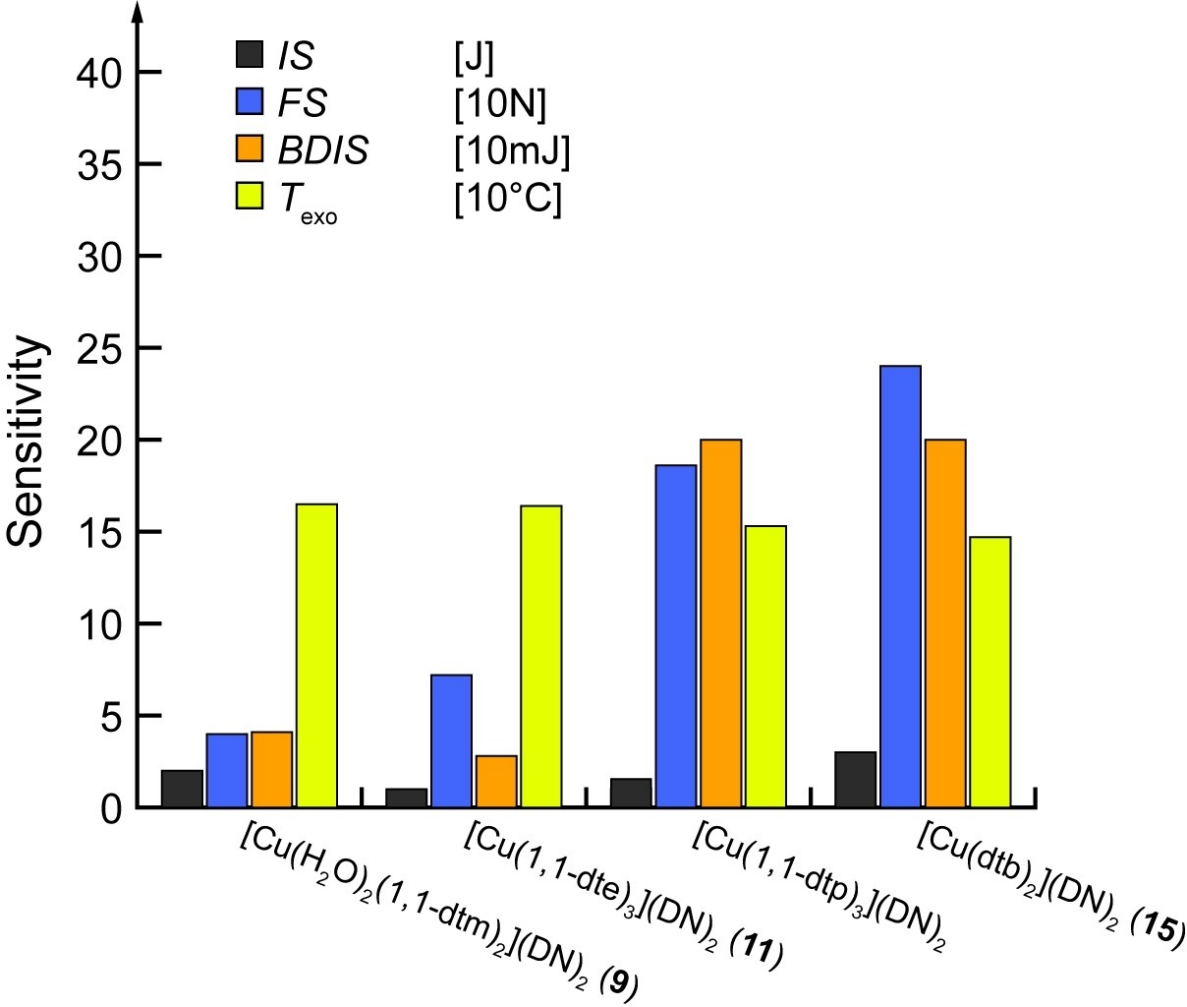
Graphical representation of the thermal stability together with the sensitivity toward various mechanical stimuli of compounds **9**, **11**, **15**, and [Cu(1,1‐dtp)_3_](DN)_2_.^[9b]^

The complex possessing the highest thermal stability is the ATRI based ECC **3** (*T*_exo_=203 °C). This is due to the bridging effect of the triazole ligands, each linking between two copper(II) centers. Similar effects can be observed for complexes **4** (*T*_exo_=195 °C), **9** (*T*_exo_=165 °C), and **11** (*T*_exo_=164 °C), although less evident. The remaining complexes with a bridging ligand motif show no signs of increased thermal stability. In fact, the exothermal events of complexes **2** (*T*_exo_=179 °C) and **5** (*T*_exo_=159 °C), both based on non‐bridging ligands, are higher. On the one hand, this is due to a known effect whereby complexes based on ditetrazoles exhibit decreasing thermal stability as the alkyl chain becomes longer.^[9c]^ This effect can also be observed in the comparison of the ditetrazoles prepared in this work (Figure 12). On the other hand, effects such as the low decomposition temperature of the ligand itself (ECC **10**), or the lower stability of the dehydrated species (compound **13**) are reasons for lower thermal stabilities.^[14]^ Nevertheless, the exothermic decomposition temperature of compound **3** clearly proofed, that a tuning of the compounds’ thermal stability is possible through the choice of the suitable ligand.

In addition, every compounds’ sensitivity toward mechanical stimuli according to BAM standard methods^[28]^ and electrostatic discharge were investigated and ranked in accordance with the UN recommendations on the transport of dangerous goods (Table 2).^[29]^ Interestingly, complex **8** is one the most sensitive complex studied in terms of its sensitivity toward friction (*FS*=3 N). This is surprising, because usually the presence of water in energetic materials leads to desensitization, which seems to be equalized by the strong endothermic character of the ligand.^[13]^ Similar sensitivities were observed for complexes **8**, **9**, and **10**. The di(tetrazolyl)methane ligands (1,1‐dtm & 1,2‐dtm) used in these complexes are known to form powerful but sensitive coordination compounds, which is why ECC **10** is the third most sensitive (*FS*=15 N), closely followed by coordination compound **9** (*FS*=40 N).^[14]^ The high friction sensitivity of the compound is unusual, as noted for complex **8**, because of the two aqua ligands. Regarding the other coordination compounds containing water ligands, it becomes clear that these are the least sensitive compounds besides complex **15** (*FS*=240 N). The high stability of the latter is due to the complex's high carbon content compared to the other ones. Also, worth mentioning is complex compound **6**, which is the second most sensitive of the compounds tested regarding friction sensitivity or thermal stability (*T*_exo_=106 °C; *FS*=5 N, *IS*=2 J). The reason for this is probably the unusual structure of the complex and its anionic dinitramido ligands, which are bonded to the central metal via a nitrogen atom. Since this is the first case of a complex showing such coordination geometry known to the authors, a proof of this assumption by literature data is unfortunately not possible at this time.

**Table 2:** Overview of the compounds’ sensitivities toward various stimuli.

	No.	*IS* (J)^[a]^	*BDIS* (mJ)^[b]^	*FS* (N)^[c]^	*ESD* (mJ)^[d]^
**[Cu(DN)_2_(NH_3_)_4_]**	**(2)**	2	>200	50	1080
**[Cu(ATRI)_3_](DN)_2_**	**(3)**	8	20	80	14
**[Cu(BTRI)_2_(H_2_O)_2_](DN)_2_**	**(4)**	7	83	144	181
**[Cu(MTZ)_6_](DN)_2_**	**(5)**	8	>200	120	203
**[Cu(AET)_2_(DN)_2_]**	**(6)**	2	28	5	250
**[Cu(DN)_2_(MAT)_4_] ⋅ 2 H_2_O**	**(7)**	4	>200	80	>1500
**[Cu(AMT)_4_(H_2_O)](DN)_2_**	**(8)**	2	28	3	250
**[Cu(1,1‐dtm)_2_(H_2_O)_2_](DN)_2_**	**(9)**	2	41	40	>1500
**[Cu(1,2‐dtm)_3_](DN)_2_**	**(10)**	2	28	15	270
**[Cu(1,1‐dte)_3_](DN)_2_**	**(11)**	≤1	28	72	76
**[Cu(2,2‐dte)_3_](DN)_2_**	**(12)**	2	25	30	250
**[Cu(H_2_O)_2_(idtp)_2_](DN)_2_**	**(13)**	2	180	120	317
**[Cu(DN)_2_(1,2‐dtp)_2_]**	**(14)**	2	>200	60	840
**[Cu(dtb)_3_](DN)_2_**	**(15)**	3	>200	240	>1500

[a] Impact sensitivity acc. to the BAM drop hammer (method 1 of 6).[^28^, ^30^] [b] Ball drop impact sensitivity (method 1 of 6) acc. to MIL‐STD 1751 A (method 1016).^[31]^ [c] Friction sensitivity acc. to the BAM friction tester (method 1 of 6).[^28^, ^32^] [d] Electrostatic discharge sensitivity (OZM Electric Spark XSpark10, method 1 of 6).^[31b]^

The effect of the carbon content on the properties of a complex, especially the stability against mechanical stimuli, can be illustrated using the example of dinitramide complexes investigated in this work. Together with the complex already published by Szimhardt *et* *al*., the complete series of copper dinitramide ECC based on ditetrazole ligands from di(tetrazol‐1‐yl)methane to 1,4‐di(tetrazol‐1‐yl)butane is known.^[9b]^

The trends shown in Figure 12 represent a fundamental concept of ECC. As the length of the carbon chain increases, sensitivity toward impact and friction decreases, whilst thermal stability drops. However, the effect of the different coordination geometry in compound **9**, compared to the other complexes, should be noted. A comparison of the sensitivity to electrostatic discharge is less meaningful since this is extremely dependent on the grain size. Ball drop impact sensitivity of [Cu(1,1‐dtp)_3_](DN)_2_ was determined and impact and friction sensitivity were redetermined using the same batch to reduce grain size influences.

Apart from the trend shown in Figure 12, there are no other significant sensitivity patterns with respect to impact sensitivity. According to the UN recommendations on the transport of dangerous goods, every compound except **3**, **4**, **5**, and **7** are ranked as very sensitive regarding impact sensitivity.^[29]^ The remaining compounds must only be considered as sensitive. In terms of sensitivity toward friction, compounds **3** and **7** are also considered very sensitive, with ECC **6** and **8** being even extremely sensitive. The complexes **13** and **15** are only considered sensitive.

**Figure 13:**
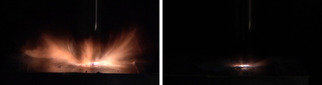
High‐speed image of the moment of detonation of complexes **6** (left) and **9** (right) during the ball drop impact test.

In addition to BAM sensitivities, the ball drop impact sensitivity (BDIS) according to MIL‐STD 1751 A (method 1016) was investigated.^[31a]^ The aim was to obtain measured values which are more realistic than data obtained mainly with the BAM drop hammer. This method is known to suffer from some weaknesses, for example, the ignition of the substance by forming hotspots within the shells.^[33]^ In the ball drop test, however, a steel ball with a defined spin is dropped onto an unconfined sample, spread over a thin layer of defined height (Figure 13).^[31a]^

Experiments were carried out for every coordination compound. The outcome is displayed in Table 2. The sensitivities obtained for compounds **5** (*IS*=8 J, *BDIS*>200 mJ, *FS*=120 N), **6** (*IS*=2 J, *BDIS*=28 mJ, *FS*=5 N), **7** (*IS*=2 J, *BDIS*>28 mJ, *FS*=3 N), **13** (*IS*=2 J, *BDIS*=180 mJ, *FS*=120 N), and **15** (*IS*=3 J, *BDIS*>200 mJ, *FS*=240 N) are either very low, or very high, which is consistent with our earlier findings.^[34]^ It is assumed that there is a strong correlation between low sensitivity toward friction and ball drop impact. However, in the case of compounds **2**, **3**, and **11** the results do not match our previous results. ECC **3** (*IS*=8 J, *BDIS*=20 mJ, *FS*=80 N) was not considered sensitive during BAM sensitivity measurements but was tested the most sensitive compound during ball drop testing. Whilst the tetrammine complex **2** (*IS*=2 J, *BDIS*>200 mJ, *FS*=50 N) was much more insensitive than expected the ball drop impact sensitivity on **11** (*IS*=1 J, *BDI*S=28 mJ, *FS*=72 N) correlates more with the BAM impact rating than with the friction sensitivity. This shows that the system of ball drop impact sensitivity has not yet been completely understood and that there are other substances that need to be investigated using this method in order to further expand our knowledge about this method and sensitivities of energetic materials in general. For safety reasons, different and unknown sensitivity values from external devices must be evaluated carefully.

A comparison of the complexes of this work with earlier work of our group on this area is difficult in most cases. Since strongly coordinating anions such as fulminate, azide, or various trinitrophenolates lead to significantly different coordination geometries, a comparison here is almost impossible. Therefore, the best way of making meaningful comparisons is probably with other oxidizing, weakly coordinating anions such as nitrate, chlorate, bromate or perchlorate. In the case of complexes **2**, **4**, **6**, **9**, **12**, and **15**, this is also not possible, since either no similarly structured complexes are known in the literature or have not been sufficiently investigated for their energetic parameters. In the cases in which a comparison was useful, it was found that apart from compound **11**, which is 0.5 J more sensitive than the respective chlorate and perchlorate complexes, all dinitramide based ECCs are less sensitive than the analogous chlorate, bromate or perchlorate complexes. The only suitable nitrate complex [Cu(MTZ)_6_](NO_3_)_2_ on the other side is less sensitive than ECC **5**.

### Primary explosive suitability evaluation

**Figure 14:**
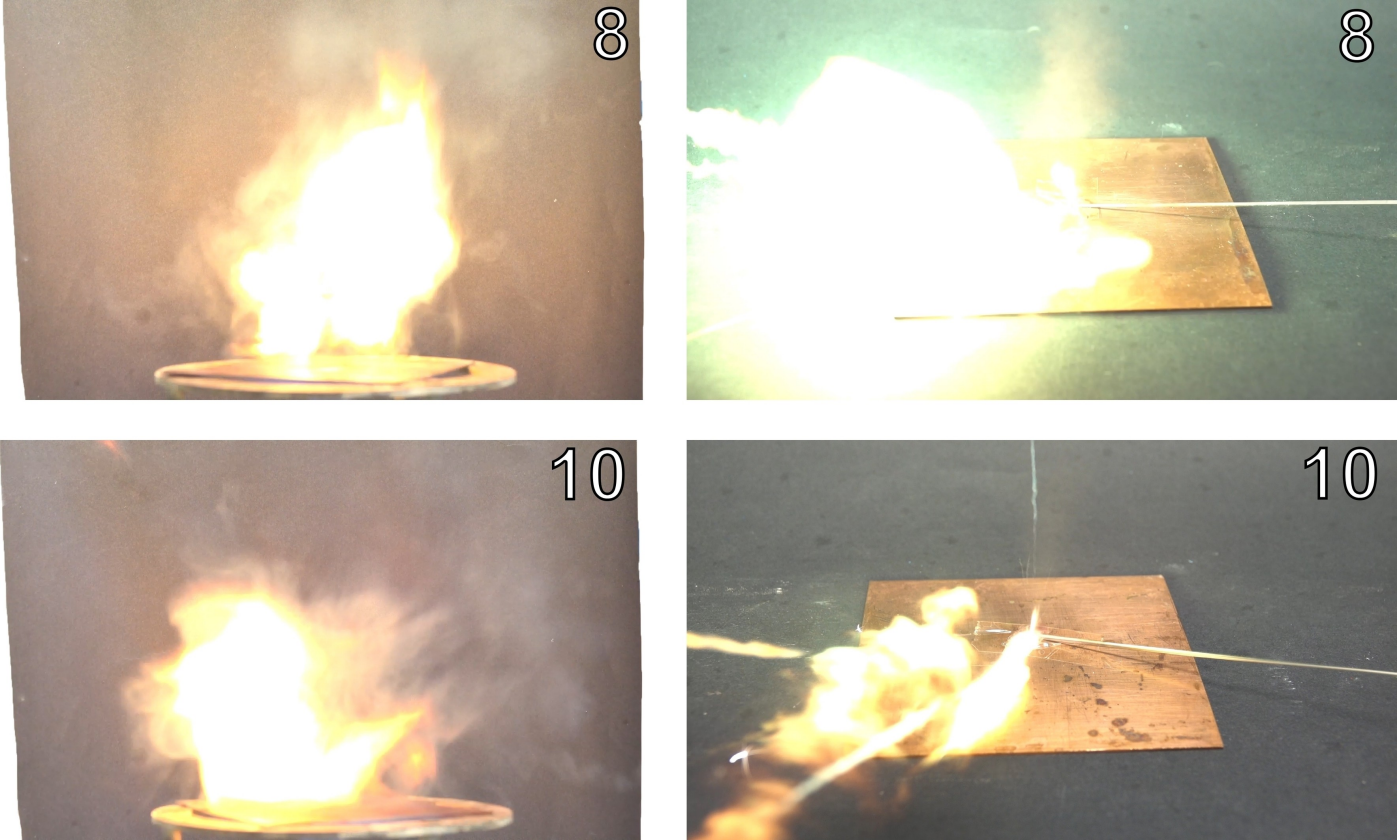
High‐speed images of deflagration reactions of compounds **8** and **10** during the hot plate test (left) and detonation in the hot needle test (right).

Hot plate and hot needle tests were performed as an initial test to get an insight into each compounds’ deflagration to detonation transition (DDT), which is a very important property of primary explosives. Investigations on DDT allow conclusions to be drawn on how well a primary is capable of initiating a booster charge such as PETN. The hot plate test displays the behavior of a compound during fast heating (Figure 14, left). Hot needle tests indicate the performance of a sample during slight confinement (Figure 14, right).

Details on the process and further high‐speed images of the experiments can be found in the General Methods in the Supporting Information (Figures S7–S19). The outcome of each test is summarized in Table 3. With the exception of **2**, **3**, **4**, and **15**, all ECC examined showed at least a deflagration in both initial experiments. These are very promising findings and the basis for further tests such as the initiation of pentaerythritol tetranitrate (PETN) or laser‐based ignitions. Since complex **8** detonated during the hot needle test and the complexes **6**, **9**, **10** and **11** showed very strong deflagrations, those compounds were investigated as lead azide (LA, Pb(N_3_)_2_) substitutes.

**Table 3:** Results of hot plate, hot needle and PETN Initiation experiments.^[a]^

	Initial Testing		PETN Initiation Experiment		Initial Testing	PETN Initiation Experiment
	HP	HN			HP	HN	
**2**	dec.	dec.	–	**9**	defl.	defl.	negative
**3**	defl.	dec.	–	**10**	defl.	defl.	negative
**4**	defl.	dec.	–	**11**	defl.	defl.	negative
**5**	defl.	defl.	–	**12**	defl.	defl.	–
**6**	defl.	defl.	negative	**13**	defl.	defl.	–
**7**	defl.	defl.	–	**14**	defl.	defl.	–
**8**	defl.	det.	positive	**15**	dec.	dec.	–

[a] –: not tested, dec.: decomposition, defl.: deflagration, det.: detonation.

**Figure 15:**
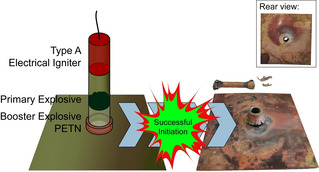
Positive initiation test result of compound **8**, together with a graphical representation of the test setup used.

To carry out the initiation experiments, 200 mg of PETN were pressed into a copper shell and initiated with 50 mg of the substance to be investigated using a Type A electric igniter. A positive result, as observed in the case of ECC **8**, is indicated by a hole in the copper plate (Figure 15). This clearly proved that by choosing the appropriate ligand, the power of the complex can be increased to a level capable of initiating PETN. A test of compound **11** indicated that this compound is close to being capable of initiating PETN. A modification of the test, for example, a variation of the particle size or quantity, could resolve this problem. Further details on the procedure can be found in the General Methods of the Supporting Information.

### Laser ignition experiments

Besides the classical ignition methods by flame, as used in initiation experiments, or by mechanical stimuli, as found in percussion caps, more and more research is performed in the field of alternative ignition methods. One of these fields where ECC are already established are laser‐based ignitions.^[16]^ Especially regarding the dinitramide anion as part of ECC, there is evidence that copper(II) complexes based on this system respond to laser radiation.[^9a^, ^9b^, ^9c^]

The principle of laser‐based ignition of primary explosives and priming mixtures has a great advantage over classical mechanical ignition. For a mechanical initiation, an impact or for primers frictional force must be generated. The substances used for this must therefore have a suitable mechanical sensitivity. For the most commonly used primary explosives lead azide (LA RD‐1333) and lead styphnate (LS) these sensitivities were determined to 0.45–1 N and 7–8 J (LS) as well as ≤0.1 N and 4 J (LA RD‐1333).[^34^, ^35^] For explosives ignited by laser irradiation, however, less sensitive substances are also suitable, which drastically reduces the risk potential during production, processing, and storage of the respective energetic materials.

**Figure 16:**
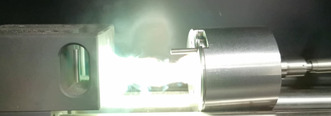
Deflagration of [Cu(MTZ)_6_)](DN)_2_ (**5**) during laser irradiation.

In this work, every compound was investigated for its behavior when irradiated with a laser beam. The outcome of each test, together with the applied energy, is shown in Table 4. Further information on the test setup and procedure can be found in the General Methods in the Supporting Information. With the exception of compound **12**, all complexes showed a reaction toward the laser irradiation. The result of such examination, in this case the result of the testing of compound **5**, is shown in Figure 16. Further high‐speed recordings of all compounds can be found in the Supporting Information (Figures S20–26).

**Table 4:** Results of laser ignition experiments.

	Laser Ignition Experiments, *E* (mJ)^[a,b]^					Laser Ignition Experiments, *E* (mJ)^[a,b]^		
	4.5	30	51	126		30	51	67.5
**2**	–	–	dec.	–	**9**	defl.	–	–
**3**	–	–	defl.	defl.	**10**	defl.	–	–
**4**	–	–	dec.	–	**11**	–	defl.	defl.
**5**	defl.	–	defl.	–	**12**	dec.	–	–
**6**	–	defl.	–	–	**13**	–	dec.	dec.
**7**	–	defl.	–	–	**14**	dec.	–	–
**8**	–	det.	–	–	**15**	defl.	–	–

[a] Operating parameters: current *I*=7–15 A; voltage *U*=4 V; theoretical maximal output power *P*_max_=45 W; theoretical energy *E*_max_=4.5–126 mJ; wavelength *λ*=915 nm; pulse length *τ*=1–15 ms. [b] –: not tested, dec.: decomposition, defl.: deflagration, det.: detonation.

The outcome of each investigation differed significantly due to the applied ligand. Thus, especially complexes **2**, **3**, **13**, **14**, and **15** showed only weak reactions, which, considering the respective hot plate and hot needle tests of the substances, is within the expectations. Coordination compounds **5**, **6**, **8**, **9**, and **10**, which are based on ligands known to form powerful complexes, showed significantly stronger reactions when irradiated by the laser, up to a detonation in the case of compound **8**. However, based on these expectations, compound **12** in particular disappointed in terms of output upon laser irradiation.

As mentioned earlier, the complex based on 2,2‐dte did not respond at all to radiation despite promising hot plate and hot needle tests. Only minimal decomposition was detected in the primer cap after completion of the test, with a bulk of the complex remaining unreacted. This is worth mentioning because complex **11**, based on the isomeric ligand 1,1‐dte, which has the same molecular formula, underwent strong deflagration during laser irradiation. Assuming that the decomposition of the sample in the laser is thermal in nature, the two‐step slow decomposition observed during the DTA measurement could be a reason for the behavior (Figure S9).

Except for compound **12**, these results suggest a possible use as potential laser‐ignitable explosives and demonstrates again that by utilizing the concept of ECC, the performance of the dinitramide anion in complexes can be adjusted by selecting the appropriate ligand system. As the mechanisms behind laser ignition (most probably thermal ignition) are still not fully understood, UV‐Vis spectra were recorded in the solid state for selected compounds. Particular emphasis was placed on the wavelength range corresponding to that of the laser when evaluating the spectra. Details on this, as well as all spectra, can be found in the Supporting Information (Table S4, Figure S27–S28).

## Conclusion

Dinitraminic acid (HDN, **1**) was successfully prepared by ion exchange techniques using an acidified Amberlite IR 120 and an aqueous ADN solution as eluent. The dinitraminic acid was further reacted with copper(II) carbonate for the *in* *situ* preparation of highly clean copper(II) dinitramide. The combination of the obtained solution with different ligands led to 13 new copper complexes of which nine were crystallographically investigated. To the best of our knowledge, it is also the first time that the tetraammine complex of the copper dinitramide has been isolated elemental pure. The complexes were analyzed in detail regarding their thermal and mechanical stability as well as their sensitivity to electrostatic discharge. The thermal stability could thus be increased to over 200 °C (**3**) using the bridging and commercially available 4‐amino‐triazole ligand. Only three compounds were less thermally stable than ADN. The systematic series of ditetrazole ligands from a methylene to a butylene bridge in copper dinitramide complexes was also completed by this work. Here, with the lengthening of the alkyl chain, a nearly halving of the sensitivity was observed. This trend contributes significantly to the concept of ECC to estimate the properties of the following complexes. In addition, all complexes were investigated in laser initiation experiments. During these, only one complex did not show a response to the irradiation, whereas compound **4** even deflagrated at an energy of 51 mJ and **8** detonated at an energy of 30 mJ. Furthermore, the concept of ECC was successfully applied to increase the compounds performance, leading to one complex (**8**) even being capable of initiating PETN. Furthermore, ditetrazole‐based complexes **11**–**15** should be considered for further testing as burn rate catalysts due to their low water solubility and decomposition temperature of about 150 °C.

## Conflict of interest

The authors declare no conflict of interest.

## Supporting information

As a service to our authors and readers, this journal provides supporting information supplied by the authors. Such materials are peer reviewed and may be re‐organized for online delivery, but are not copy‐edited or typeset. Technical support issues arising from supporting information (other than missing files) should be addressed to the authors.

Supplementary
