# Supplementary material for: A Smart Access to the Dinitramide Anion – The Use of Dinitraminic Acid for the Preparation of Nitrogen‐Rich Energetic Copper(II) Complexes
Source: Chemistry. 2021 Jun 10;27(35):9112–23. doi: 10.1002/chem.202100747 (PMC8362218; doi:10.1002/chem.202100747)
Supplement: Supplementary file 1 — Supplementary [file CHEM-27-9112-s001.pdf]

Chemistry–A European Journal

Supporting Information

A Smart Access to the Dinitramide Anion – The Use of Dinitraminic Acid for the Preparation of Nitrogen-Rich Energetic Copper(II) Complexes

Michael S. Gruhne, Maximilian H. H. Wurzenberger, Marcus Lommel, and Jörg Stierstorfer*

Author Contributions

M.G. Investigation:Lead; Writing – original draft:Lead
M.W. Methodology:Equal; Writing – review & editing:Lead
M.L. Data curation:Lead; Software:Lead

Supporting Information

Table of Contents

1. Compounds Overview
2. Single Crystal X-Ray Diffraction
3. IR Spectroscopy of **2**–**15**
4. DTA Plots of **2**–**15**
5. TGA Plots of **2**, **4**–**9**, **13**, **15**
6. Hot Plate & Hot Needle Tests of **2**–**15**
7. Laser Ignition Experiments of **2**–**15**
8. UV-Vis Spectroscopy of **2**–**15**
9. Experimental Part and General Methods
10. References

1. Compounds Overview

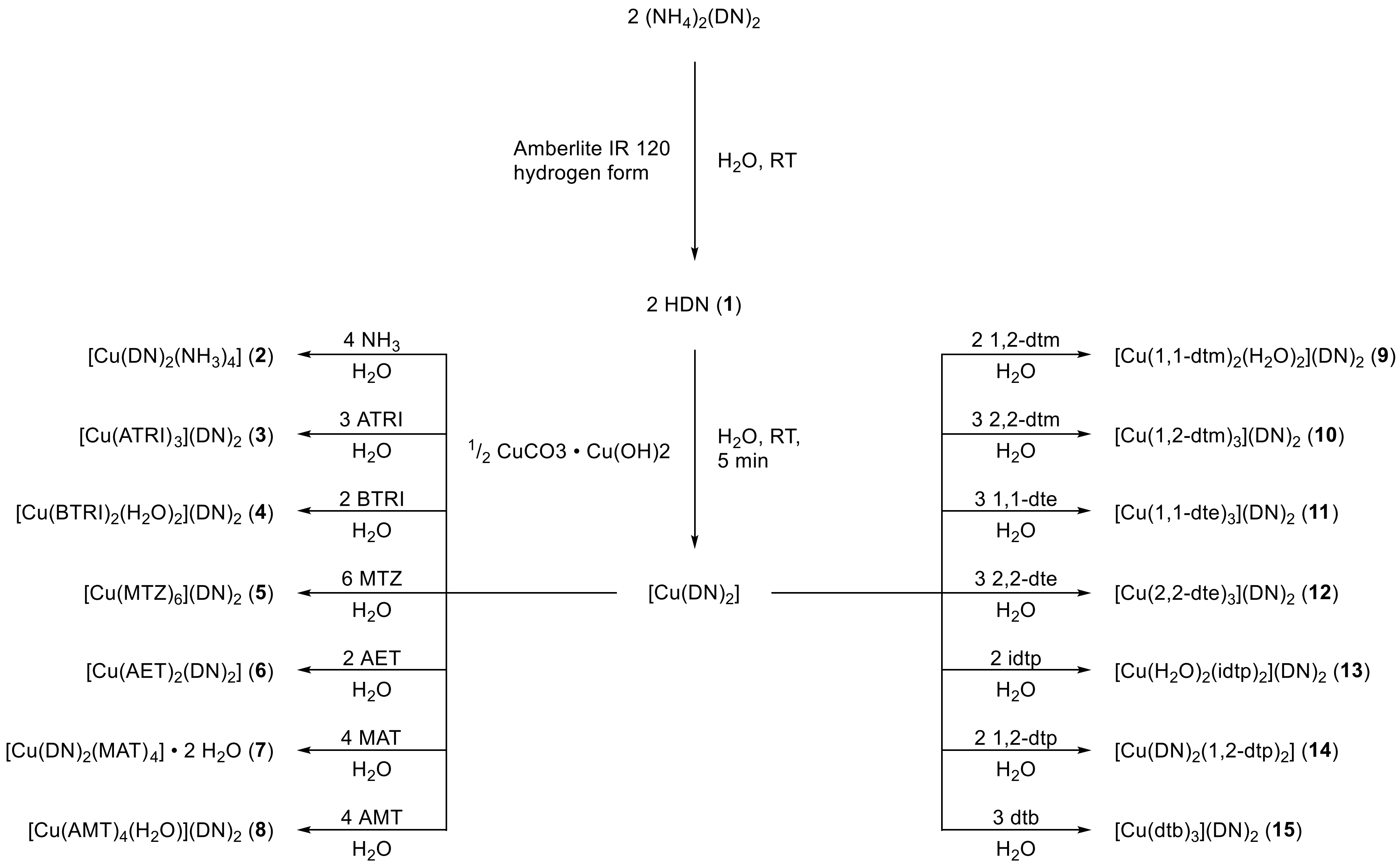

2. Single Crystal X-Ray Diffraction

For all crystalline compounds, an Oxford Xcalibur3 diffractometer with a CCD area detector or Bruker D8 Venture TXS diffractometer equipped with a multilayer monochromator, a Photon 2 detector and a rotating-anode generator were employed for data collection using Mo-K_α radiation (λ = 0.7107 Å). On the Oxford device, data collection and reduction were carried out using the CRYSALISPRO software.[S1] On the Bruker diffractometer, the data were collected with the Bruker Instrument Service v3.0.21, the data reduction was performed using the SAINT V8.18C software (Bruker AXS Inc., 2011). The structures were solved by direct methods (SIR-92,[S2] SIR-97,[S3] SHELXS-97[S4] or SHELXT[S5]) and refined by full-matrix least-squares on *F*2 (SHELXL[S4]) and finally checked using the PLATON software[S6] integrated in the WinGX[S7] or Olex2[S8] software suite. The non-hydrogen atoms were refined anisotropically and the hydrogen atoms were located and freely refined. The absorptions were corrected by a SCALE3 ABSPACK or SADABS Bruker APEX3 multi-scan method.[S9,S10] All DIAMOND2 plots are shown with thermal ellipsoids at the 50% probability level and hydrogen atoms are shown as small spheres of arbitrary radius.

Table S1. Crystallographic data of the coordination compounds **4**, **6**, and **8**.

	**4**	**6**	**8**
Formula	$C_8H_{12}CuN_{18}O_{10}$	AET	$C_8H_{22}CuN_{26}O_9$
FW [g mol^{-1}]	583.90		690.05
Crystal system	monoclinic		monoclinic
Space group	$P2_1/n$		$P2_1/n$
Color / Habit	blue block		Blue plate
Size [mm]	0.12 x 0.15 x 0.23		0.01 x 0.03 x 0.05
a [Å]	6.5409(4)		15.4651(12)
b [Å]	12.8112(6)		18.1488(14)
c [Å]	11.9052(6)		20.5886(17)
α [°]	90		90
β [°]	92.107(5)		110.062(3)
γ [°]	90		90
V [Å^3]	996.94(9)		5428.0(8)
Z	2		8
$\rho_{calc.}$ [g cm^{-3}]	1.945		1.689
μ [mm^{-1}]	1.195		0.897
F(000)	590		2824
$\lambda_{MoK\alpha}$ [Å]	0.71073		0.71073
T [K]	120		173
θ Min-Max [°]	3.4, 26.4		2.0, 26.4
Dataset	−8: 8 ; −16: 16 ; −14: 14		−19: 19 ; −22: 22 ; −24: 25
Reflections collected	7572		97637
Independent refl.	2029		11113
R_{int}	0.034		0.089
Observed reflections	1849		8184
Parameters	177		816
R_1 (obs)[a]	0.0395		0.0647
wR_2 (all data)[b]	0.1023		0.1797
GooF[c]	1.10		1.03
Resd. Dens. [e Å^{-3}]	−0.33, 1.14		-0.60, 1.26
Absorption correction	multi-scan		multi-scan
Device type	Oxford Xcalibur3		Bruker D8 Venture TXS
CCDC			

a) $R_1 = \Sigma||F_0|-|F_c||/\Sigma|F_0|$; b) $wR_2 = [\Sigma[w(F_0^2-F_c^2)^2]/\Sigma[w(F_0)^2]]^{1/2}$; $w = [\sigma c^2(F_0^2)+(xP)^2+yP]^{-1}$ and $P = (F_0^2+2F_c^2)/3$; c) GooF = $\{\Sigma[w(F_o^2-F_c^2)^2]/(n-p)\}^{1/2}$ (n = number of reflections; p = total number of parameters).

Table S2. Crystallographic data of the compounds **10–11**.

	**9**	**10**	**11**
Formula	$C_6H_{12}CuN_{22}O_{10}$	$C_9H_{12}CuN_{30}O_8$	$C_{12}H_{18}CuN_{30}O_8$
FW [g mol^{-1}]	615.92	732.03	774.10
Crystal system	monoclinic	triclinic	triclinic
Space group	$P2_1/n$	$P{-}1$	$P{-}1$
Color / Habit	blue block	blue block	blue block
Size [mm]	0.04 x 0.05 x 0.12	0.19 x 0.41 x 0.50	0.08 x 0.12 x 0.17
a [Å]	8.6435(5)	8.7831(4)	7.6855(6)
b [Å]	9.4591(7)	9.5477(5)	8.7967(6)
c [Å]	12.4210(9)	16.3487(8)	11.5671(9)
α [°]	90	88.425(4)	90.040(6)
β [°]	92.819(6)	83.104(4)	109.044(7)
γ [°]	90	70.693(4)	95.627(6)
V [Å^3]	1014.31(12)	1284.40(11)	735.21(10)
Z	2	2	1
$\rho_{calc.}$ [g cm^{-3}]	2.017	1.893	1.748
μ [mm^{-1}]	1.186	0.956	0.840
F(000)	622	738	393
$\lambda_{MoK\alpha}$ [Å]	0.71073	0.71073	0.71073
T [K]	123	123	173
θ Min-Max [°]	2.7, 26.4	2.6, 26.4	4.3, 26.4
Dataset	−10: 10 ; −11: 11 ; −14: 15	−10: 10 ; −11: 11 ; −20: 20	−9: 9 ; −10: 8 ; −14: 14
Reflections collected	7981	18944	3965
Independent refl.	2073	5229	2968
R_{int}	0.049	0.032	0.034
Observed reflections	1591	4466	2653
Parameters	194	473	247
R_1 (obs)[a]	0.0365	0.0315	0.0441
wR_2 (all data)[b]	0.0824	0.0777	0.1084
GooF[c]	1.06	1.04	1.06
Resd. Dens. [e Å^{-3}]	−0.37, 0.47	−0.52, 0.52	−0.76, 0.81
Absorption correction	multi-scan	multi-scan	multi-scan
Device type	Oxford Xcalibur3	Oxford Xcalibur3	Oxford Xcalibur3
CCDC			

a) $R_1 = \Sigma||F_0|-|F_c||/\Sigma|F_0|$; b) $wR_2 = [\Sigma[w(F_0^2-F_c^2)^2]/\Sigma[w(F_0)^2]]^{1/2}$; $w = [\sigma c^2(F_0^2)+(xP)^2+yP]^{-1}$ and $P = (F_0^2+2F_c^2)/3$; c) GooF = $\{\Sigma[w(F_o^2-F_c^2)^2]/(n-p)\}^{1/2}$ (n = number of reflections; p = total number of parameters).

Table S3. Crystallographic data of the complexes **12**, **13**, and **15**.

	**12**	**13**	**15**
Formula	$C_{12}H_{18}CuN_{30}O_8$	$C_{10}H_{20}CuN_{22}O_{10}$	$C_{18}H_{30}CuN_{30}O_8$
FW [g mol^{-1}]	774.10	672.02	858.26
Crystal system	monoclinic	monoclinic	triclinic
Space group	*C*2/*c*	*P*2$_1$/*n*	P-1 (No. 2)
Color / Habit	blue block	blue block	blue block
Size [mm]	0.25 x 0.46 x 0.52	0.01 x 0.02 x 0.10	0.16 x 0.28 x 0.49
a [Å]	13.1564(7)	8.5187(8)	7.6842(4)
b [Å]	11.5462(6)	7.4825(7)	10.2699(8)
c [Å]	19.1139(9)	20.1102(17)	11.7236(9)
α [°]	90	90	71.446(7)
β [°]	102.722(5)	96.550(4)	87.190(5)
γ [°]	90	90	77.736(6)
V [Å^3]	2832.2(3)	1273.5(2)	856.90(11)
Z	4	2	1
$\rho_{calc.}$ [g cm^{-3}]	1.816	1.753	1.663
μ [mm^{-1}]	0.872	0.952	0.730
F(000)	1572	686	441
$\lambda_{MoK\alpha}$ [Å]	0.71073	0.71073	0.71073
T [K]	123	106	108
θ Min-Max [°]	2.2, 26.4	2.5, 26.4	2.1, 26.4
Dataset	−15: 16 ; −14: 12 ; −19: 23	−10: 10 ; −9: 9 ; −25: 25	−9: 8 ; −11: 12 ; −11: 14
Reflections collected	9176	18577	5800
Independent refl.	2884	2591	3464
R_{int}	0.033	0.082	0.023
Observed reflections	2392	2267	2988
Parameters	232	205	259
R_1 (obs)[a]	0.0342	0.0983	0.0363
wR_2 (all data)[b]	0.0846	0.2144	0.0859
GooF[c]	1.03	1.21	1.05
Resd. Dens. [e Å^{-3}]	−0.43, 0.46	−0.91, 2.49	−0.39, 0.33
Absorption correction	multi-scan	multi-scan	multi-scan
Device type	Bruker D8 Venture TXS	Bruker D8 Venture TXS	Oxford Xcalibur3
CCDC			

a) $R_1 = \Sigma||F_0|-|F_c||/\Sigma|F_0|$; b) $wR_2 = [\Sigma[w(F_0^2-F_c^2)^2]/\Sigma[w(F_0)^2]]^{1/2}$; $w = [\sigma c^2(F_0^2)+(xP)^2+yP]^{-1}$ and $P = (F_0^2+2F_c^2)/3$; c) GooF $= \{\Sigma[w(F_o^2-F_c^2)^2]/(n-p)\}^{1/2}$ (n = number of reflections; p = total number of parameters).

ECC **9** crystallizes in the form of blue platelets in the monoclinic space group $P2_1/n$. The unit cell consists of four formula units with a calculated densitiy of 1.689 g cm^{-3} at 173 K. The asymmetric unit, however, consists of two formula units built up from fivefold coordinated copper centres that differ slightly (Figure 1). Both of the square planar pyramids are slightly distorted (N9–Cu1–O1 97.86(16), N39–Cu2–O2 100.54(15)) and differ mainly in the torsion angle of the opposing AMT moieties. The ligands surrounding the center Cu1 (N3–N4–N13–N14 56.7(4), N8–N9–N19–N18 65.2(4)) are rotated further apart than the heterocycles surrounding Cu2 (N23–N24–N34–N33 52.3(4), N28–N29–N39–N38 55.2(4)). Due to te ligands steric, the top of every pyramid is occupied by an aqua ligand. As a result of a disorder, one of the dinitramid units was split.

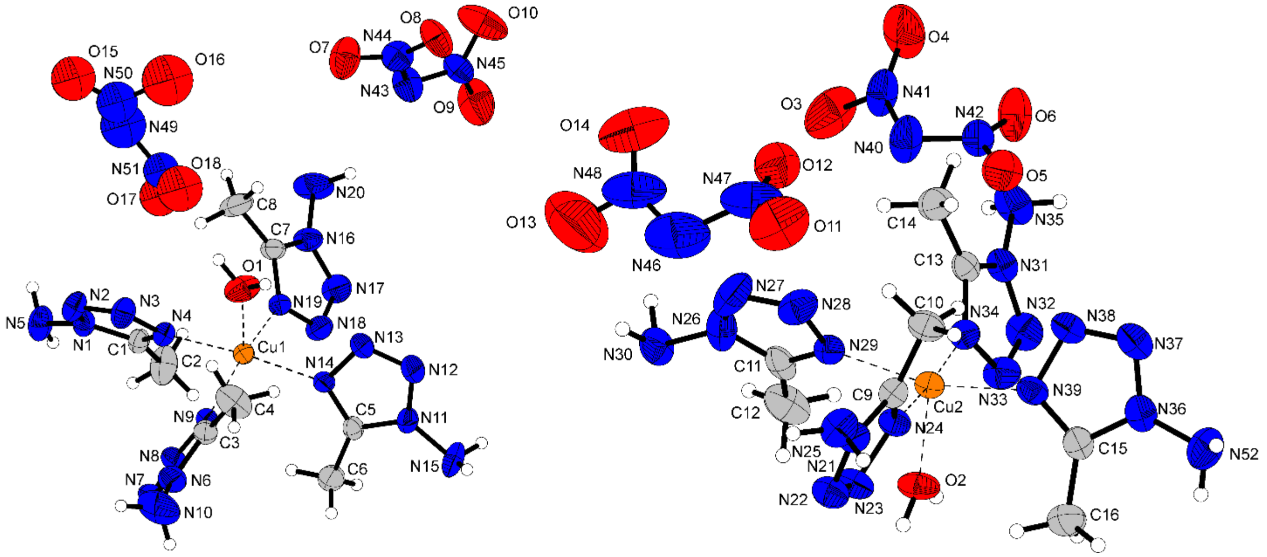

Figure 1. Separate representation of both formula units from the asymmetric unit of $[Cu(AMT)_4(H_2O)](DN)_2$ (**9**). Selected bond lengths (Å): Cu1–N4 2.006(4), Cu1–N9 2.015(4), Cu1–N14 2.003(4), Cu1–N19 2.009(4), Cu1–O1 2.154(4), Cu2–N24 2.016(4), Cu2–N29 2.018(4), Cu2–N34 2.011(4), Cu2–N39 2.018(4), Cu2–O2 2.150(3). Selected bond angles (°): N4–Cu1–O1 94.19(15), N14–Cu1–O1 94.68(15), N19–Cu1–O1 97.48(16), N24–Cu2–O2 94.42(14), N29–Cu2–O2 97.01(15), N34–Cu2–O2 93.41(14).

Coordination compound **10** crystalizes in the form of blue blocks in the triclinic space group *P*−1. A unit cell is built up by two formula units a possesses a calculated density of 1.893 g cm^{-3} at 123 K. A fomula unit consists of two different copper centers, both showing octahedral coordination environment (Figure 2). The differently coordinated copper centres are a result of how the 1,2-dtm molecules binding toward them are arranged. The ligands can be divided into two groups: while the N−C−N angle betwenn both heterocycles is only slightly different in all three ligands (N1–C2–N6 109.56(16), N9–C5–N14 110.06(17), N17–C8–N22 110.50(16)), the tetrazole rings in one of the two ligands are oriented differently to each other (C1–N1–N6–N5 12.85(18), C4–N9–N14–N13 137.23(18), C7–N17–N22–N23 119.40(18). The latter named ligands with a larger torsion angle are found in both the axial and equatorial positions. It should be noted that N2 substituted sides of the 1,2-dtm ligands are found exclusively in the axial position and the N1 substituted sides of the ligands are found exclusively in the equatorial position. The remaining equatorial coordination sites are occupied by the ligands possessing smaller C–N–N–N angles. The ligands, which have similar angles, then coordinate the same copper centres in pairs, which leads to the formation of chains. As shown in Figure 2 (right side), the remaining ligands connect these chains into two-dimensional metal-organic frameworks. Due to a disorder, one of the dinitramid moieties was split

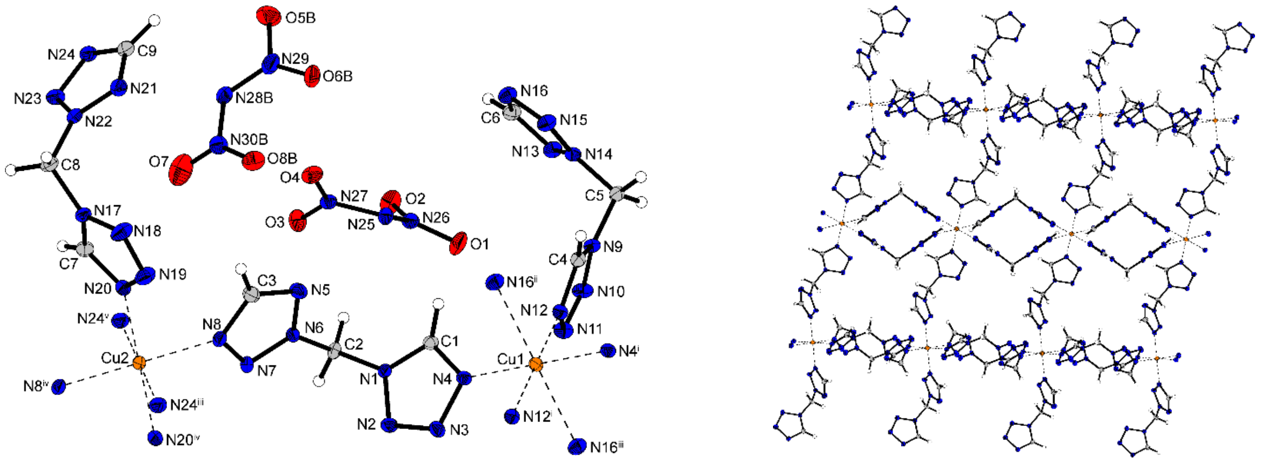

Figure 2. Coordination environment of ECC **10** (left) and polymeric network formed by 1,2-dtm ligands (right). Selected bond lengths (Å): Cu1–N4 1.9935(17), Cu1–N12 2.0327(16), Cu1–N16 2.4594(17), Cu2–N 2.0211(16), Cu2–N20 2.0371(16), Cu2–N24 2.3672(18). Selected bond angles (°): N4–Cu1–N12 88.69(7), N12–Cu1–N16 91.57(6), N4–Cu1–N16 89.03(6), N8–Cu2–N20 90.45(7), N8–Cu2–N24 95.33(7), N20–Cu2–N24 90.41(6). Symmetry codes: (i) −x, 1−y, 1−z; (ii) 1−x, 1−y, 1−z; (iii) −1+x, y, z; (iv) 1−x, −y, −z; (v) 2−x, −y, −z.

The copper (II) dinitramide complex **11** crystallizes as blue blocks in the triclinic space group *P*−1 with one formula unit per unit cell and a calculated density of 1.748 g cm^{-3} at 173 K. The symmetry is reduced to the space group *P*1 due to a disorder of the dinitramide anions. The coordination sphere consists of three tetrazole ligands bridging between different copper(II) centers (Figure 3). A Jahn-Teller distortion is observed along the N8–Cu1–N24 axis (Cu1–N8 (2.414(7) Å) and Cu1–N24 (2.447(7) Å)).

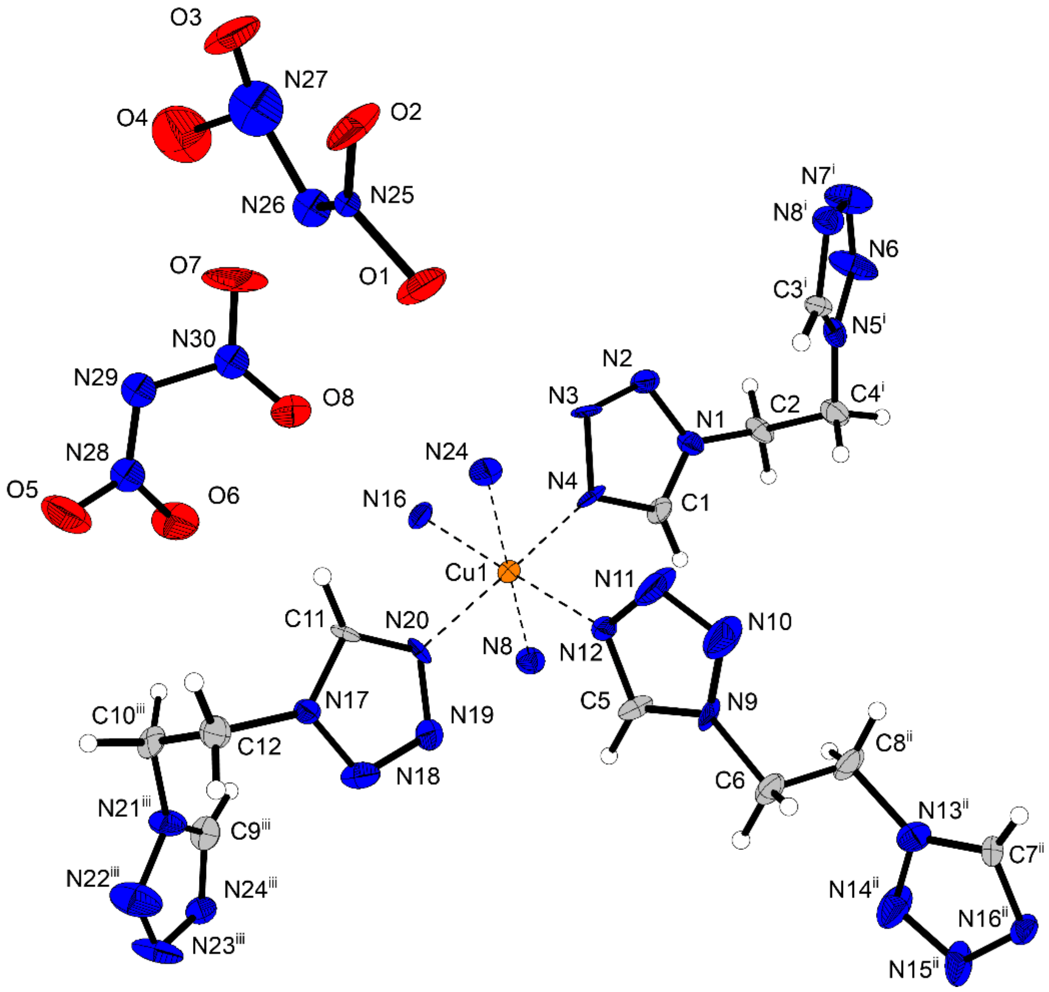

Figure 3. Coordination environment of [Cu(1,1-dte)$_3$](DN)$_2$ (**11**). Selected bond lengths (Å): Cu1–N4 1.992(7), Cu1–N12 2.026(7); selected bond angles (°): N4–Cu1–N8 89.2(3), N4–Cu1–N12 88.4(3), N4–Cu1–N20 179.4(3), selected torsion angles (°): N1–C2–C4^{i}–N5^{i} −72.3(9), N9–C6–C8^{ii}–N13^{ii} 177.7(7). Symmetry codes: (i) x, −1+y, z; (ii) 1+x, y, 1+z; (iii) x, 1+y, z.

3. IR Spectroscopy of 2–15

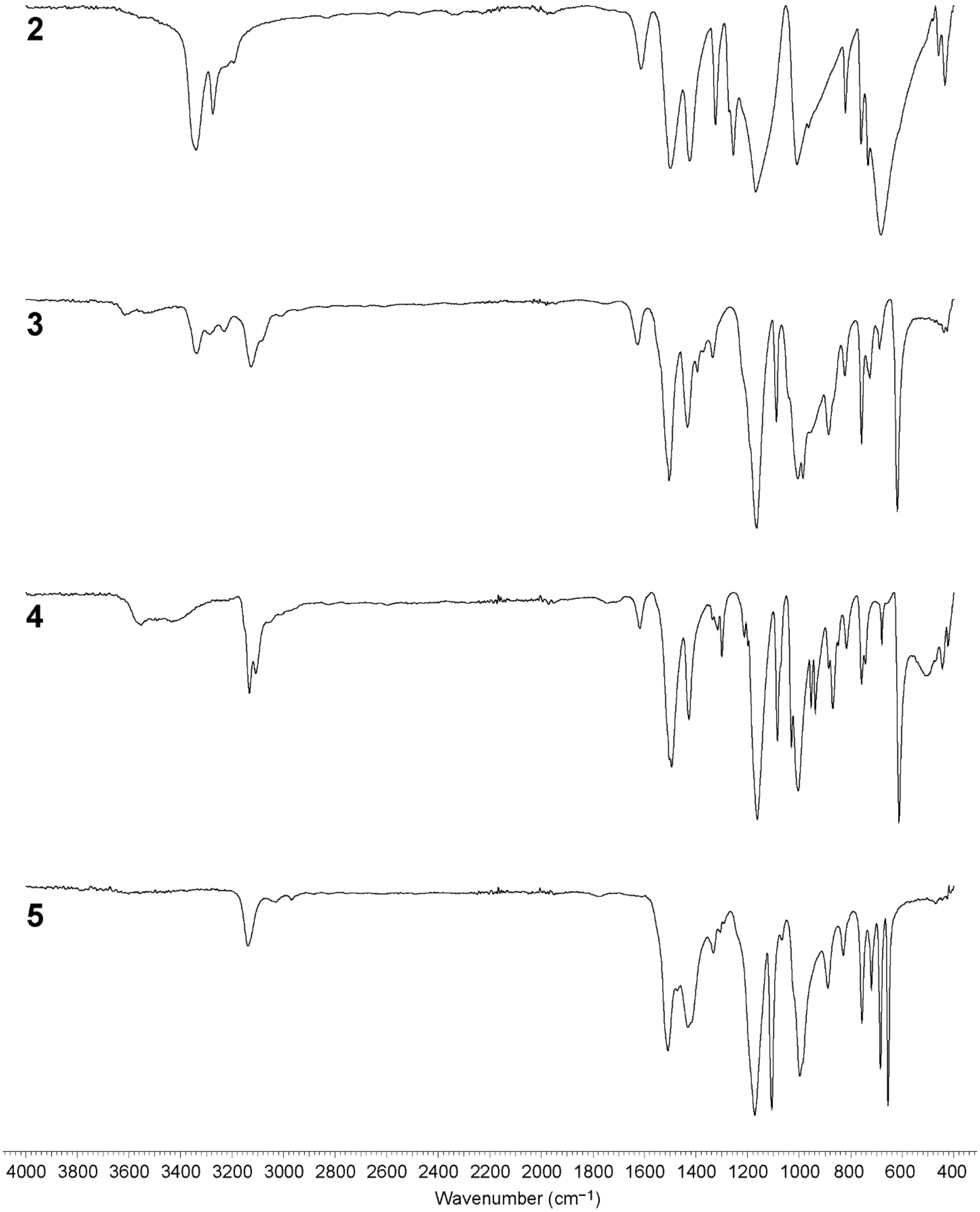

Figure S4. IR spectra of compounds **2**–**5**.

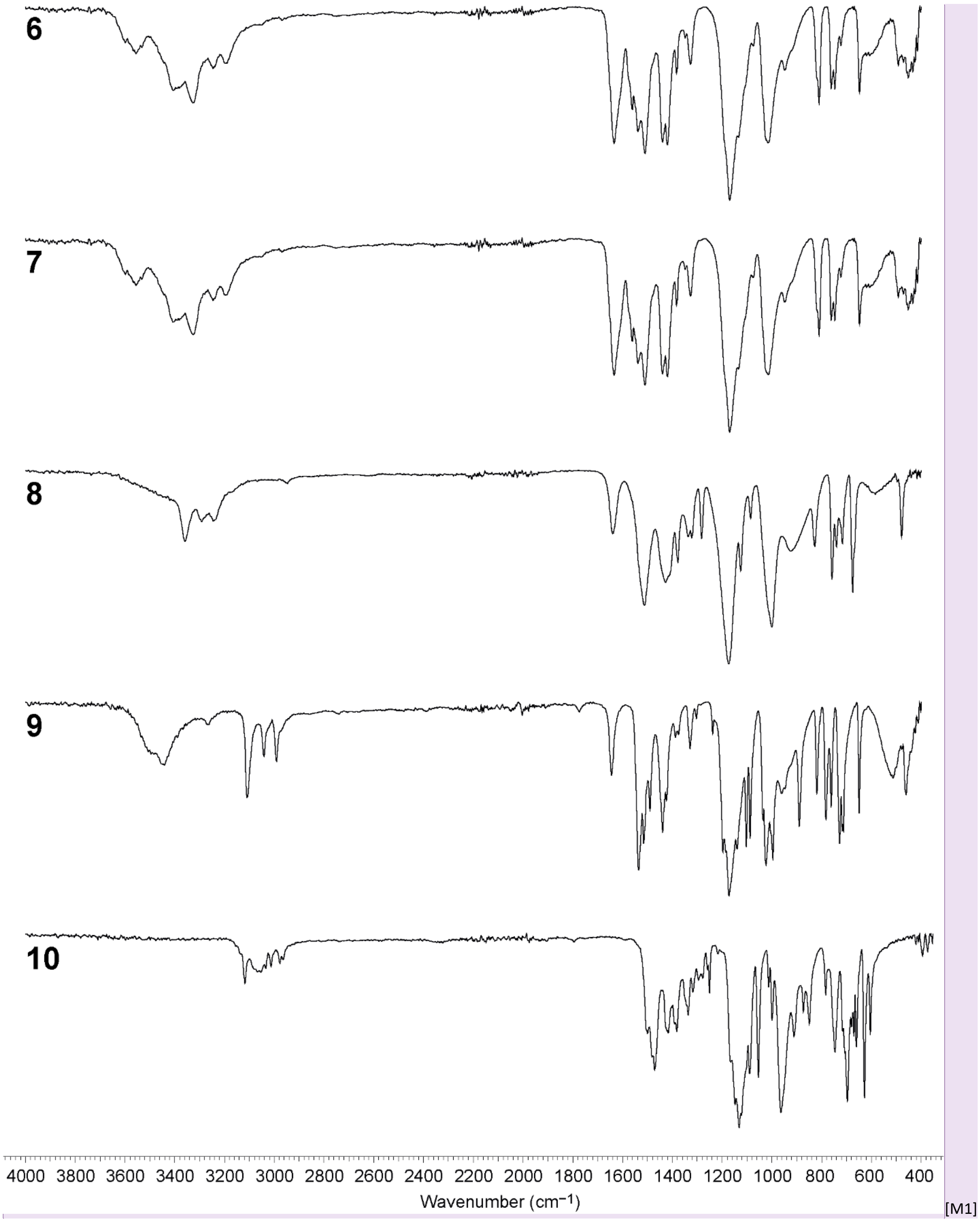

Figure S5. IR spectra of ECC **6–10**.

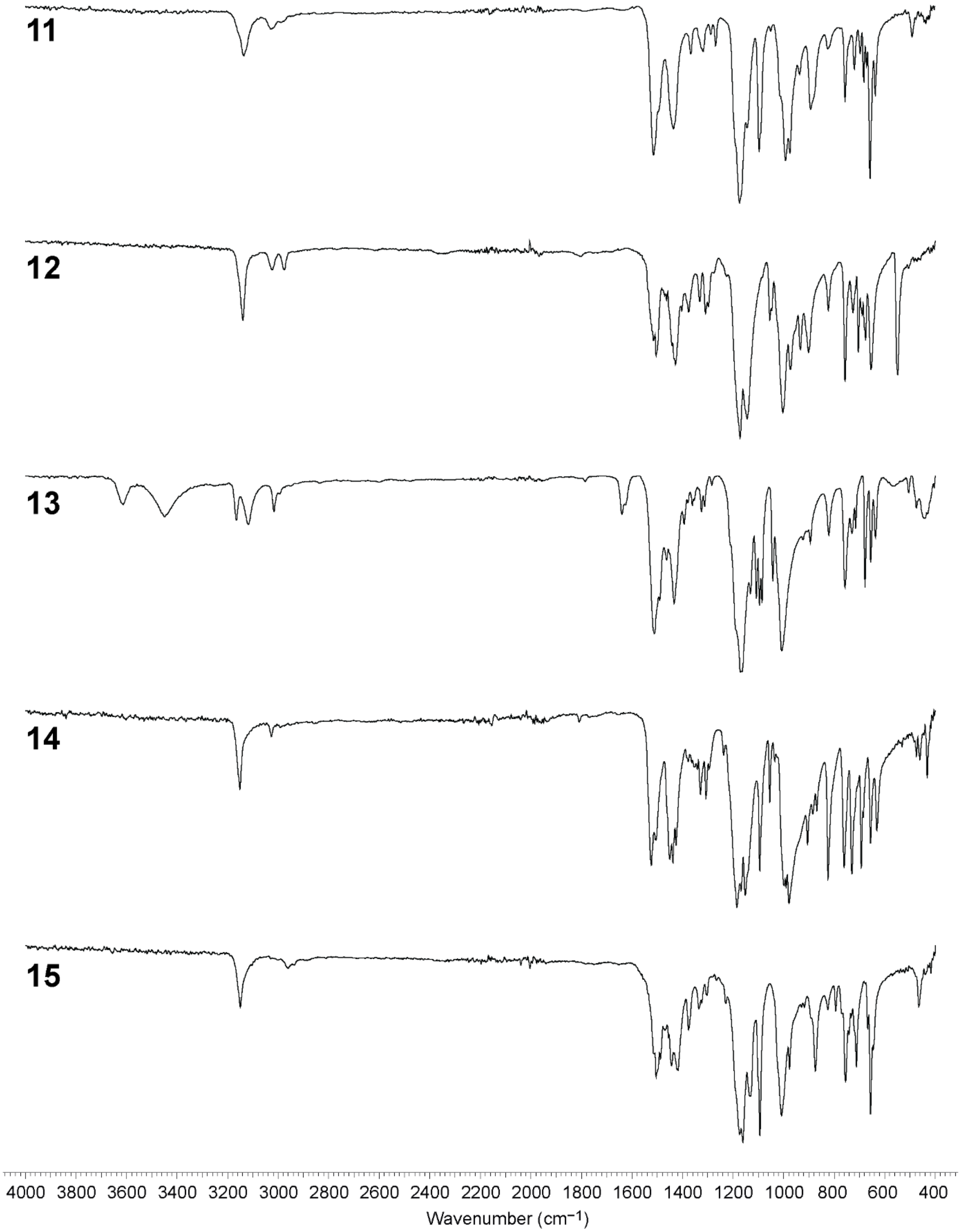

Figure S6. IR spectra of the coordination compounds **11**–**15**.

4. DTA Plots of 2–15

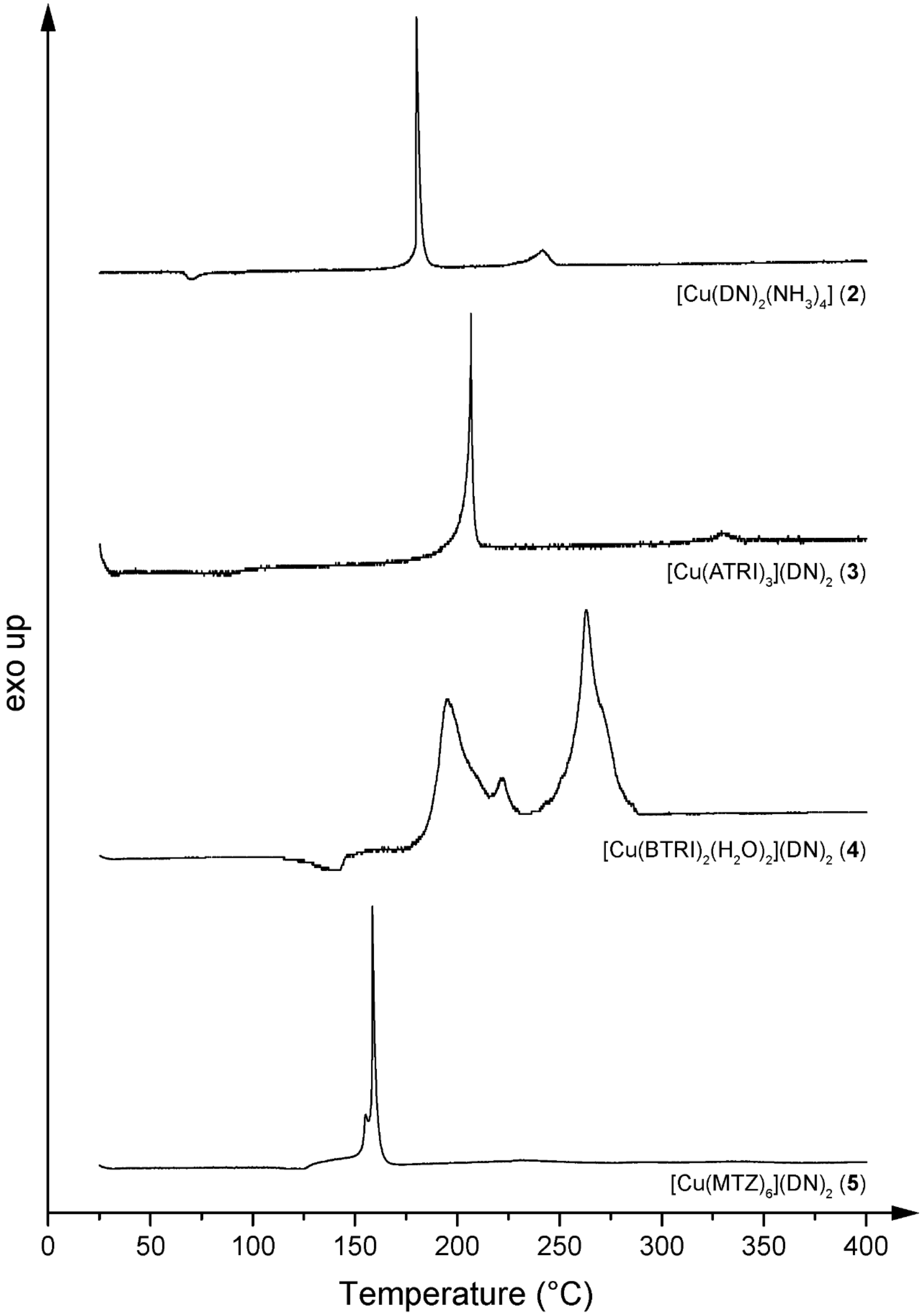

Figure S7. DTA plots of the ECC **2–5**.

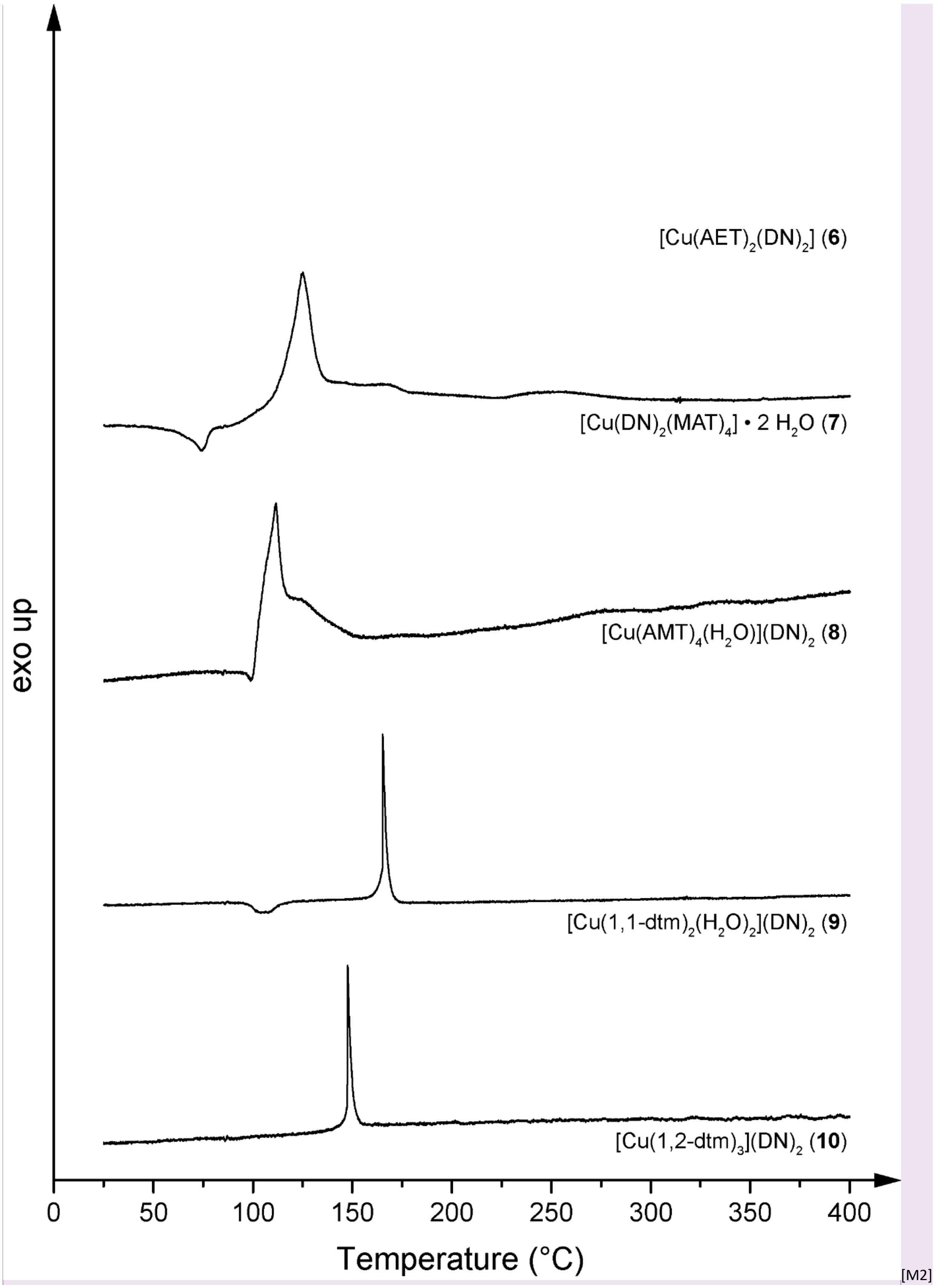

Figure S8. DTA plots of the coordination compounds **6**–**10**.

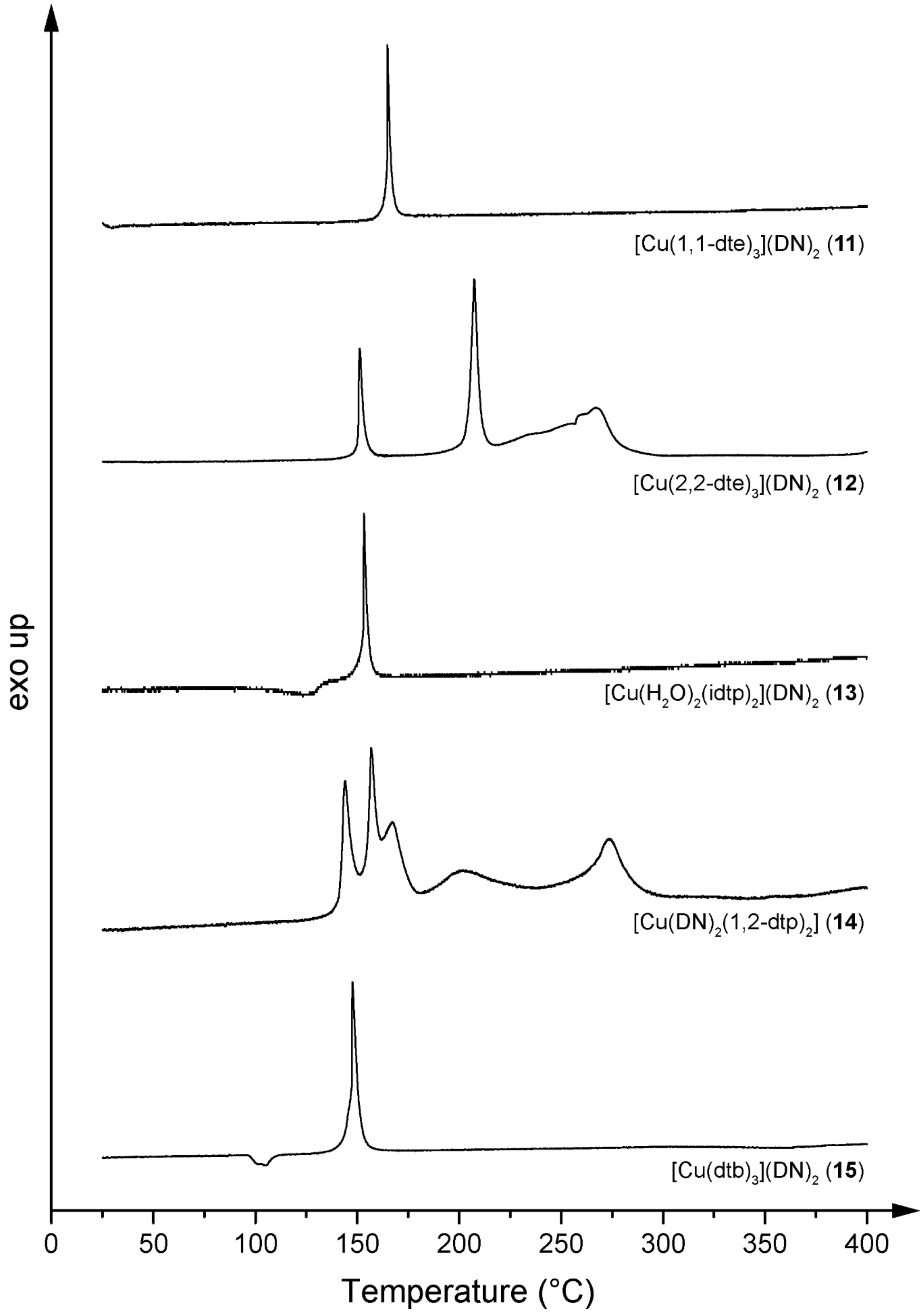

Figure S9. DTA plots of the compounds **11–15**.

5. TGA Plots of 2, 3, 5, 7–9, 13, 15

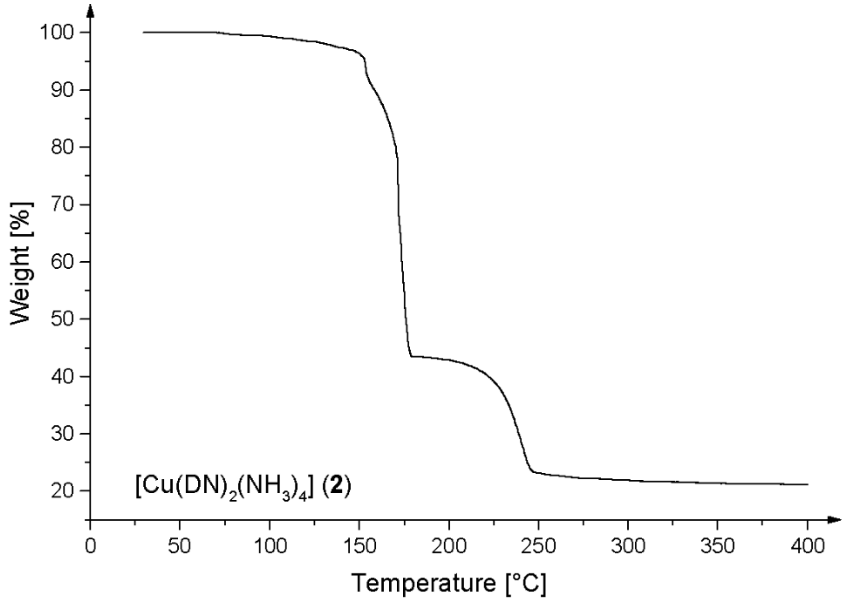

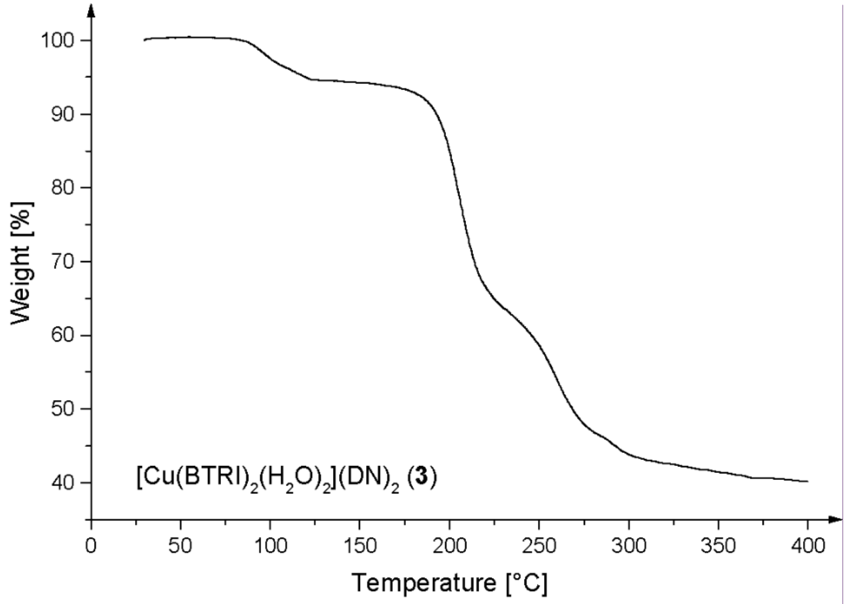

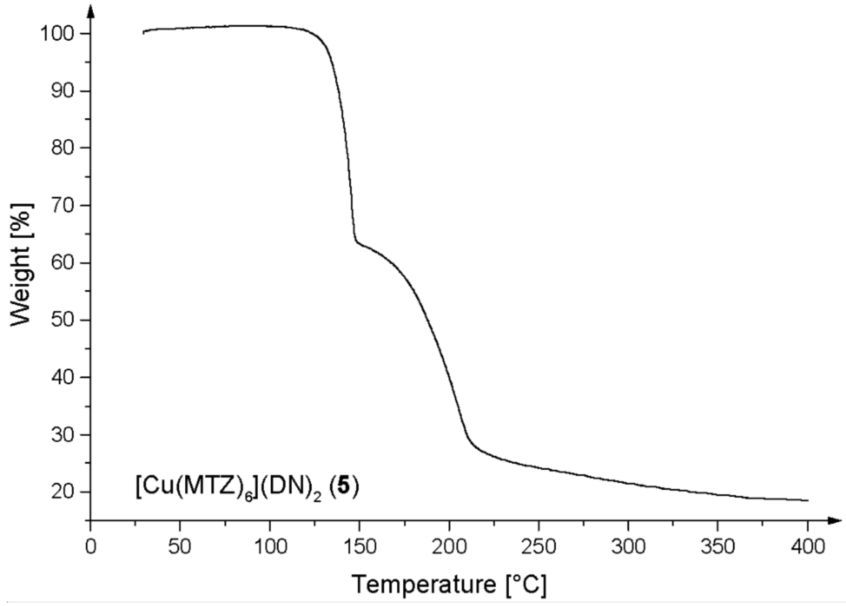

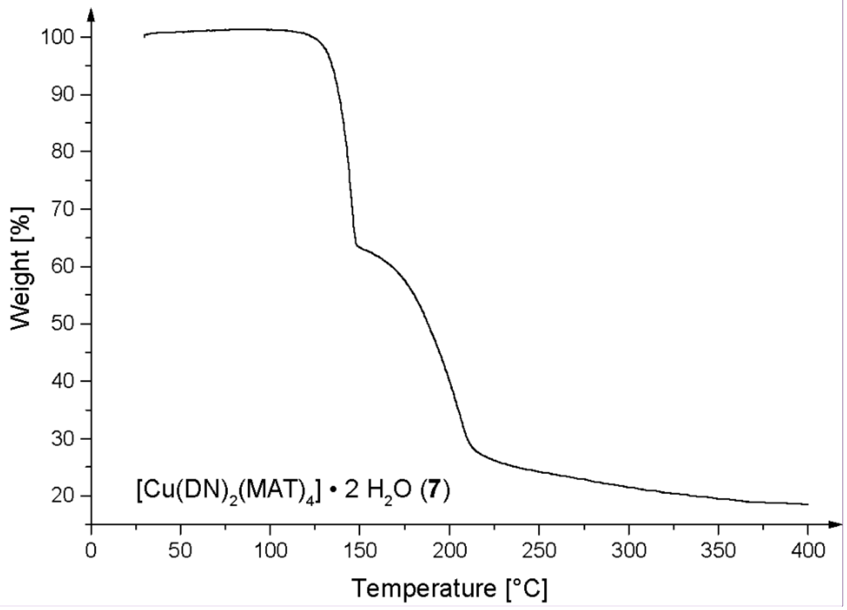

[M3]

Figure S10. TGA plots of compounds **2**, **4**, **5**, and **7**.

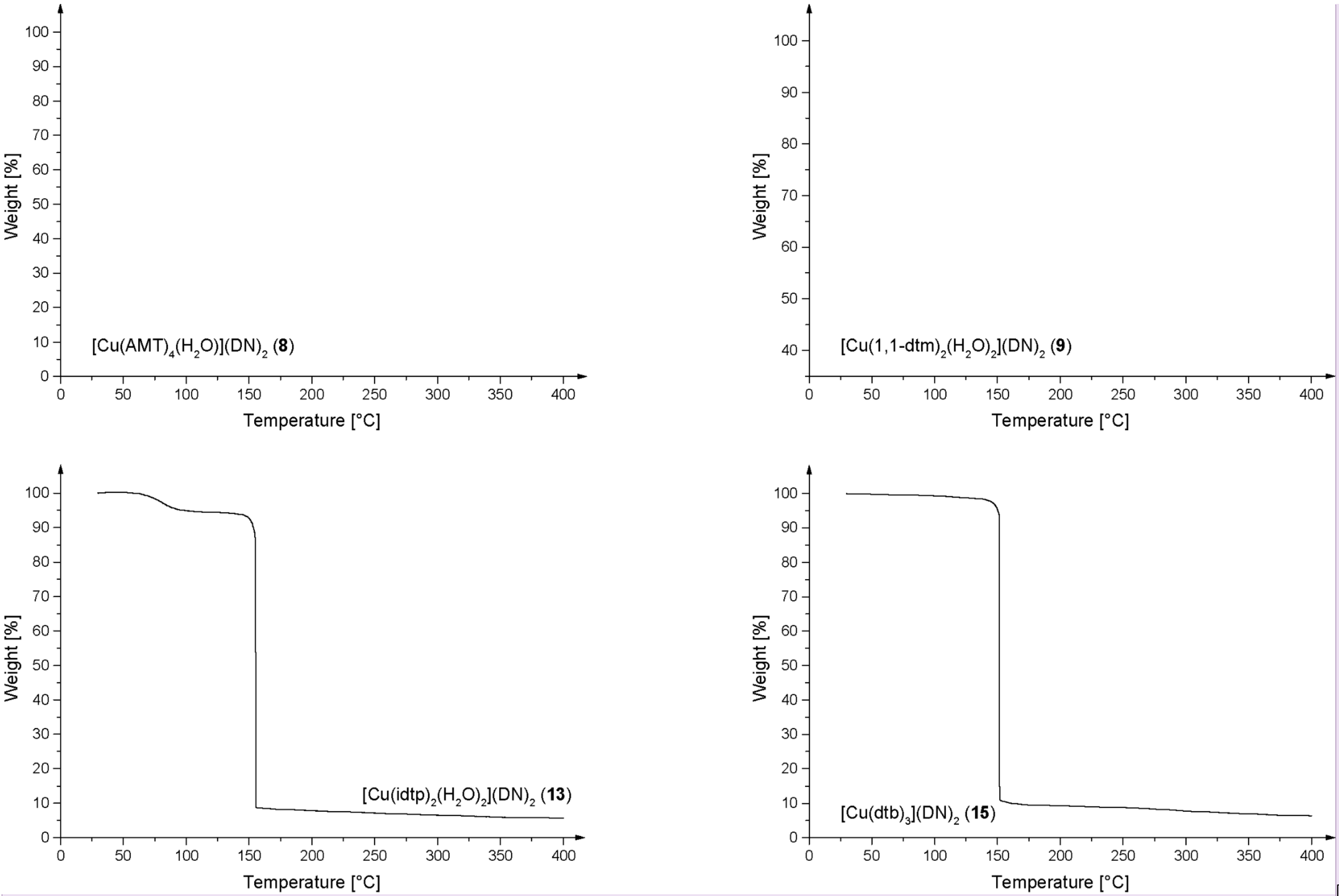

[M4]

Figure S11. TGA plots of compounds **8**, **9**, **13**, and **15**.

6. Hot Plate & Hot Needle Tests of 2–15

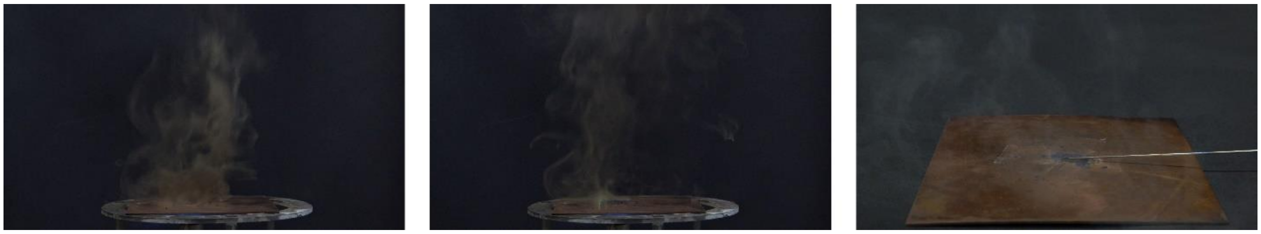

Figure S7. Decomposition reaction of compound **2** during hot plate (left & center) and hot needle test (right).

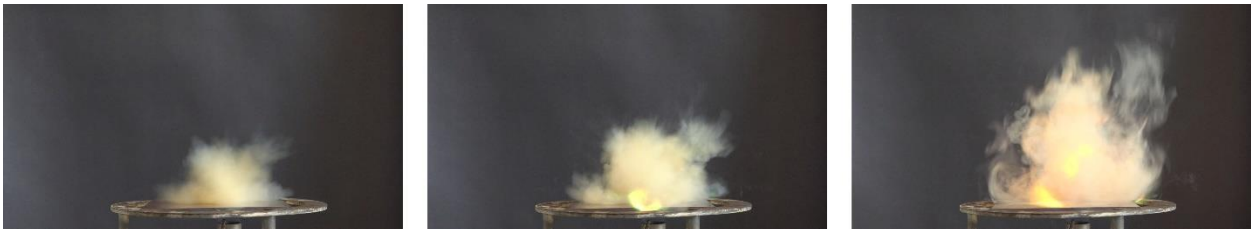

Figure S8. Deflagration of compound **3** during the hot plate test (left & center) and decomposition in the hot needle test (right).

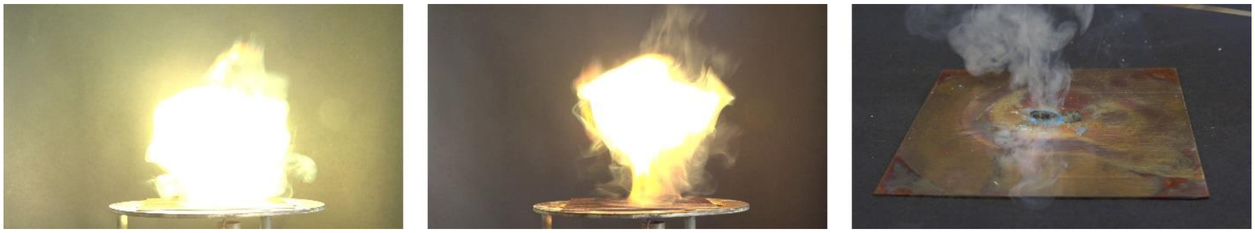

Figure S9. Behavior of compound **4** while hot plate (left & center) and hot needle testing (right).

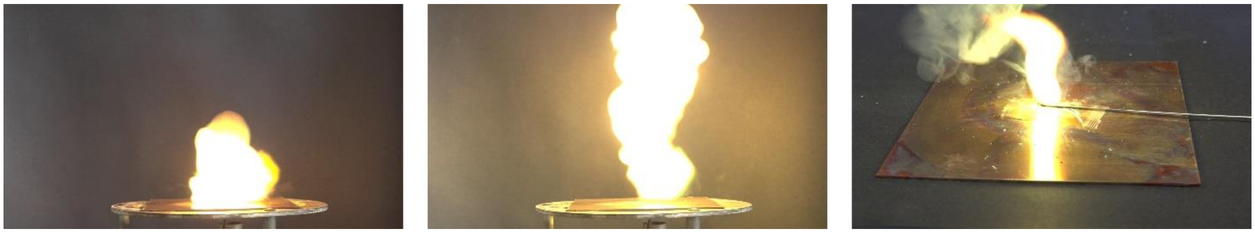

Figure S10. Hot plate (left & center) and hot needle tests (right) of coordination compound **5**.

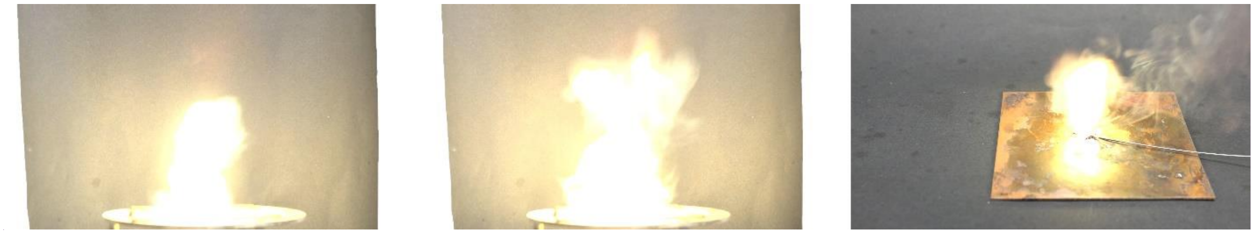

Figure S11. Deflagration of compound **7** during the hot plate test (left & center) and decomposition in the hot needle test (right).[M5]

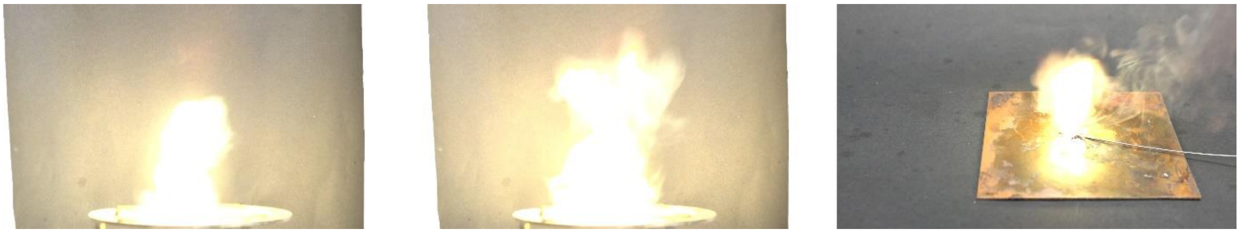

Figure S11. Deflagration of compound **7** during the hot plate test (left & center) and decomposition in the hot needle test (right).

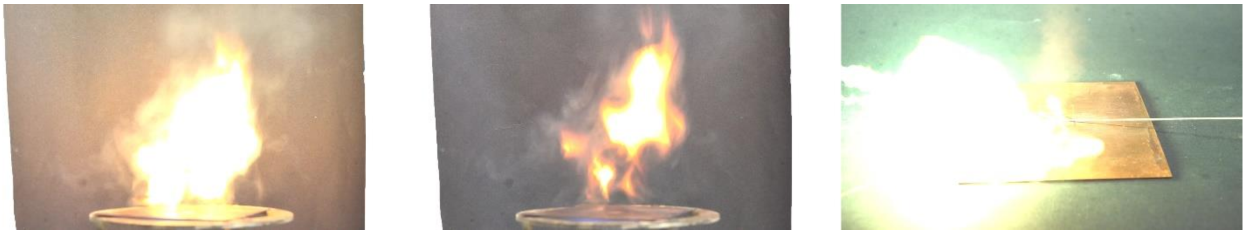

Figure S12. Deflagration reaction of compound **8** during hot plate (left & center) and detonation while hot needle test (right).

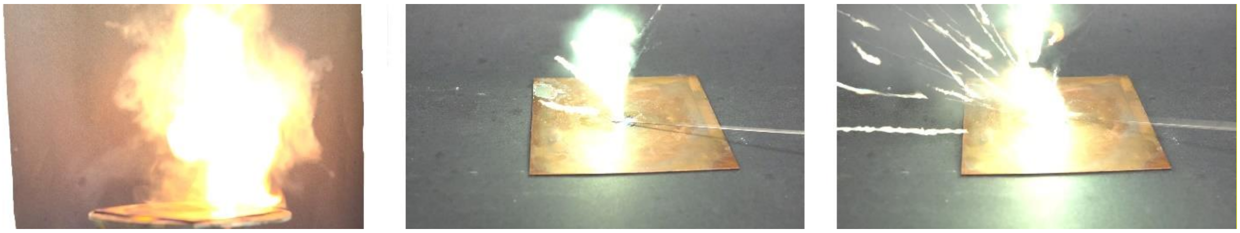

Figure S13. Reaction of compound **9** while hot plate (left) and hot needle testing (center & right).

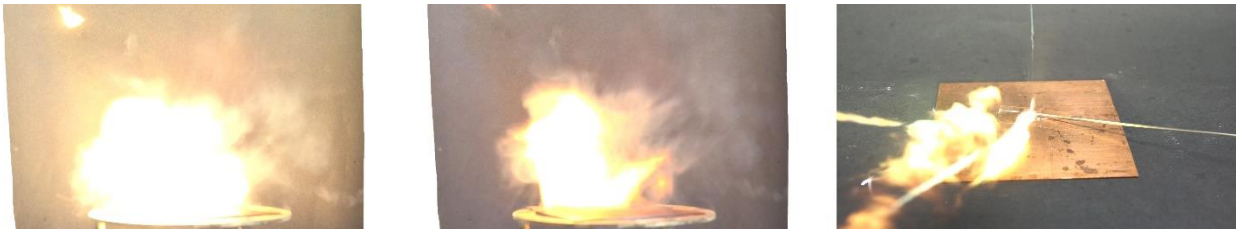

Figure S14. Hot plate (left & center) and hot needle tests (right) of coordination compound **10**.

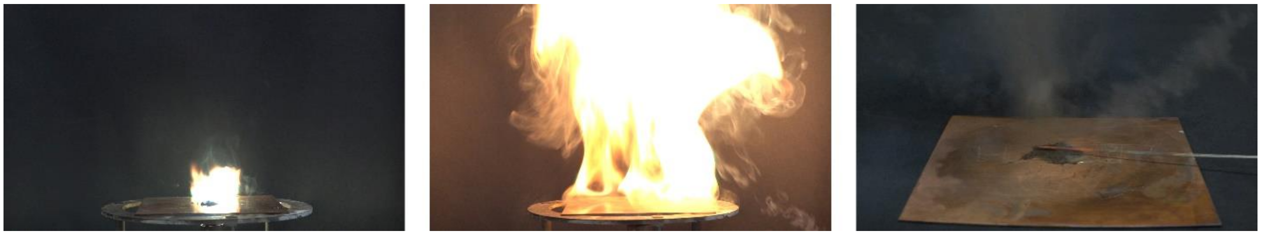

Figure S15. Behavior of compound **11** while hot plate test (left & center) and hot needle test (right).

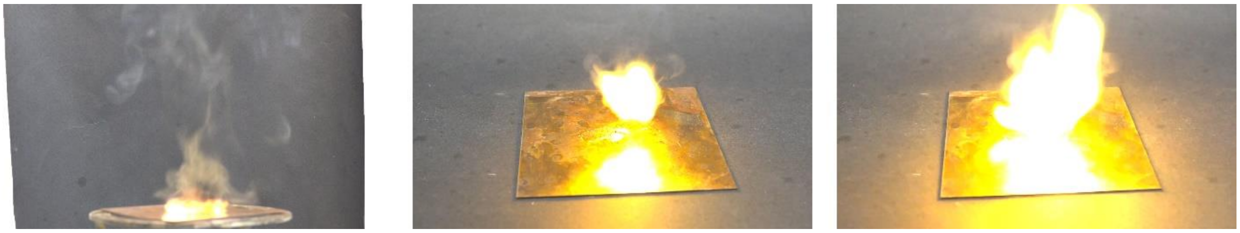

Figure S16. Deflagration reactions of compound **12** during hot plate (left) and hot needle tests (center & right).

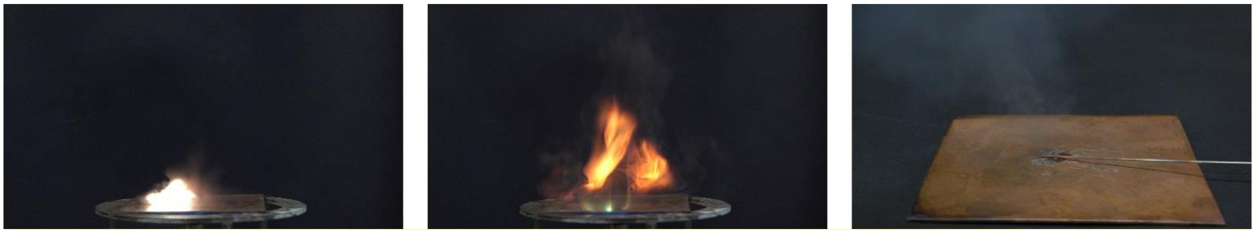

Figure S17. Reaction of compound **13** during hot plate (left & center) and hot needle testing (right).

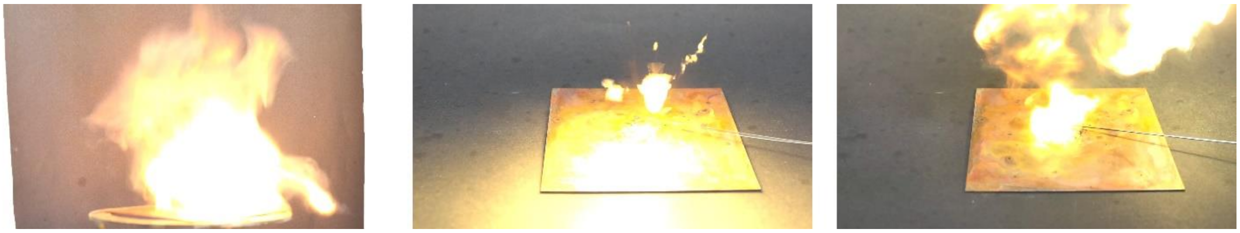

Figure S18. Hot plate (left) and hot needle tests (center & right) of coordination compound **14**.

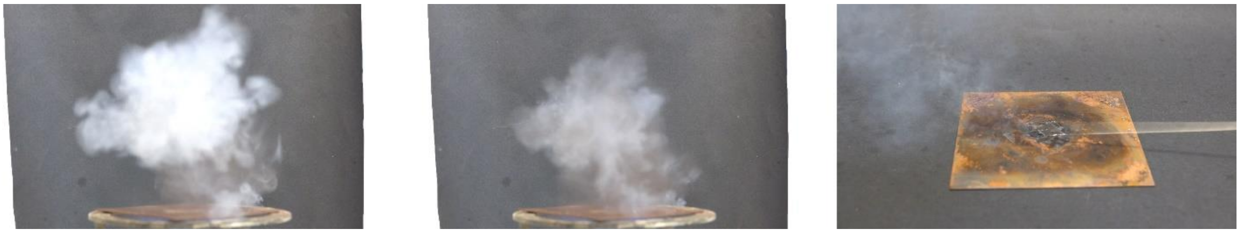

Figure S19. Decomposition reactions of compound **15** during hot plate (left & center) and hot needle tests (right).

7. Laser Ignition Experiments of 2–15

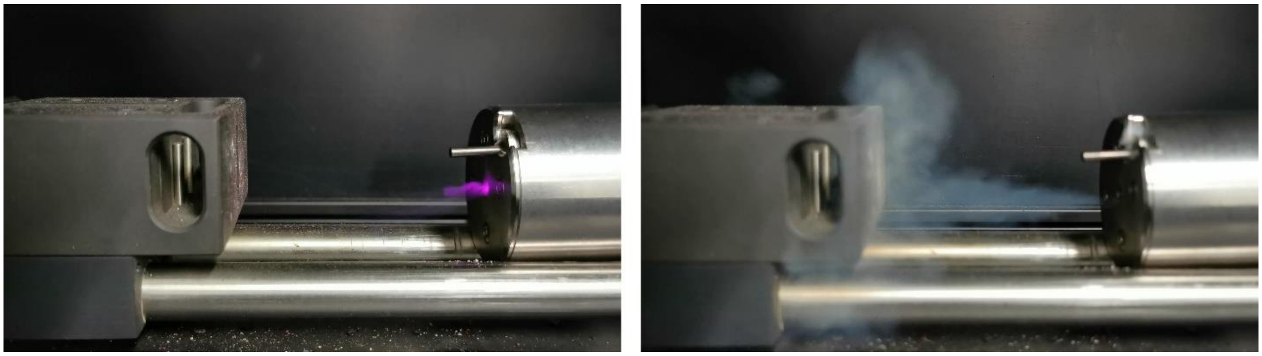

Figure S1. Decomposition of compound **16** (left, 15 ms, 12 A) and **17** (right, 15 ms, 10 A) during laser ignition experiments.

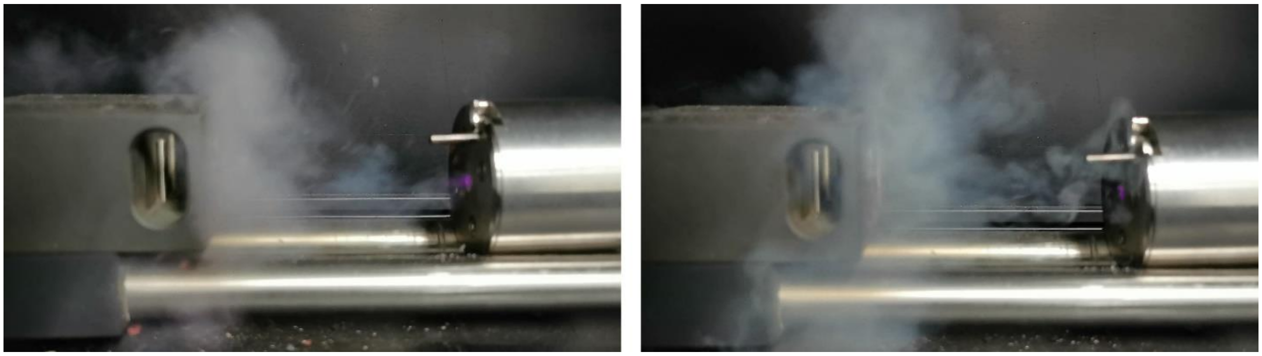

Figure S2. Deflagration of coordination compound **18** (left, 1 ms, 10 A) and **19** (right, 15 ms, 10 A) during laser ignition experiments.

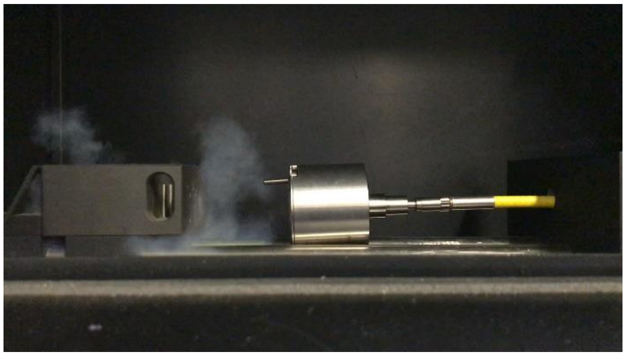

Figure S3. Deflagration of complex **20** (15 ms, 12 A).

8. UV-Vis Spectroscopy of 2–15

The process of laser initiation is still not fully understood today. For this reason, the topic has been heavily researched. In this work, solid state UV-Vis spectroscopy was performed to investigate the mechanism in more detail (Figure 10).

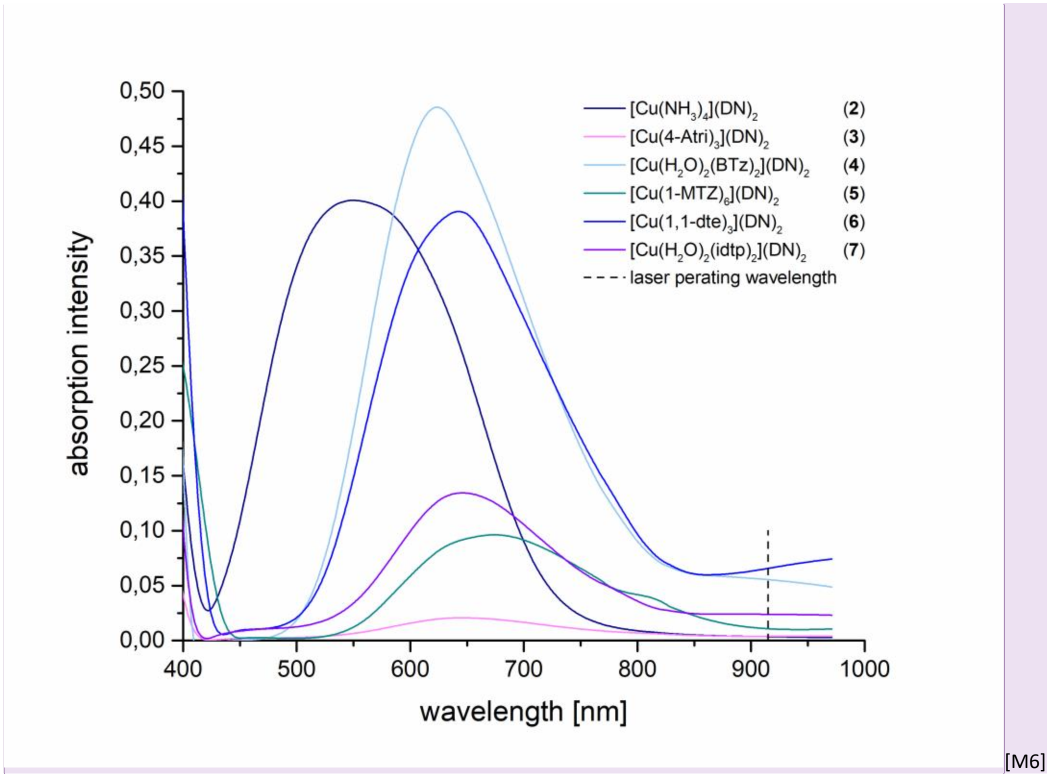

Figure 11. UV/vis spectra of the coordination compounds **2**–**15**.

Details of the optical properties can be found in Table 3. The focus is of these spectra is in the range of 915 nm, the operating wavelength of the used laser diode. The observed absorption in the near infrared and visible light is caused by the d–d transitions based on the copper center and its interaction with the dinitramide moiety and the respective ligands (Figure 10).

Table 3. Optical properties measured for the coordination compounds **2–15**.

	M	Color	λ_{d-d}[a]	$\lambda_{915}/\lambda_{d-d}$[b]		M	Color	λ_{d-d}[a]	$\lambda_{915}/\lambda_{d-d}$[b]
**2**	Cu^{II}	Blue	550	0.01	**9**				
**3**	Cu^{II}	Blue	646	0.09	**10**				
**4**	Cu^{II}	Blue	624	0.11	**11**	Cu^{II}	Blue	643	0.17
**5**	Cu^{II}	Blue	674	0.11	**12**				
**6**	Cu^{II}				**13**	Cu^{II}	Blue	646	0.18
**7**	Cu^{II}				**14**				
**8**	Cu^{II}				**15**				

[a] Absorption intensity maximum wavelength, which can be assigned to electron d–d transitions in the measured range of 350–1000 nm. [b] Quotient of the absorption intensity at the laser wavelength and the intensity at the d–d absorption wavelength.

All complexes show a moderate absorption behavior at the laser wavelength of 915 nm. This could be a possible explanation for the inflammability by laser radiation. It is known that compounds which appear colorless and show no absorption in the UV-Vis spectrum cannot be ignited by laser.Xx

9. Experimental Part & General Methods

All chemicals and solvents were employed as received (Sigma-Aldrich, Fluka, Acros, ABCR). ^{14}N spectra were recorded at ambient temperature using a JEOL Bruker 27400, Eclipse 270, JEOL EX 400 or a JEOL Eclipse 400 instrument. The chemical shifts quoted in ppm in the text refer to typical standards such as nitromethane (^{14}N) in D_2O as the solvent. Endothermic and exothermic events of the described compounds, which indicate melting, loss of aqua ligands, crystal water or decomposition, are given as the extrapolated onset temperatures. The samples were measured in a range of 25–400 °C at a heating rate of 5 °C min^{-1} through differential thermal analysis (DTA) with an OZM Research DTA 552-Ex instrument and in some cases additionally by thermal gravimetric analysis (TGA) with a PerkinElmer TGA4000. Infrared spectra were measured with neat samples on a Perkin-Elmer BXII FT-IR system with a Smith DuraSampler IR II diamond ATR. Determination of the carbon, hydrogen and nitrogen contents was carried out by combustion analysis using an Elementar Vario El (nitrogen values determined are often lower than the calculated ones' due to their explosive behavior). UV-Vis spectra were recorded in the solid state using a Varian Cary 500 spectrometer in the wavelength range of 350–1000 nm. The step in the absorption intensity at 800 nm is caused by a detector change. Impact sensitivity tests were carried out according to STANAG 4489[11] with a modified instruction[12] using a BAM (Bundesanstalt für Materialforschung) drophammer.[13,14] Ball drop impact sensitivity tests were determined for selected compounds on an OZM ball drop machine (BIT-132), following MIL-STD-1751A (method 1016) by dropping a free-falling steel ball onto the explosive compound.[13,15] Friction sensitivity tests were carried out according to STANAG 4487[16] with a modified instruction[17] using the BAM friction tester.[13,14] The classification of the tested compounds

results from the “UN Recommendations on the Transport of Dangerous Goods”.[18,19] Additionally, all compounds were tested upon the sensitivity toward electrical discharge using the OZM Electric Spark XSpark10 device.[13] Hot plate and hot needle tests were performed in order to evaluate the potential initiation capability of selected complexes. The samples were fixed on a copper plate underneath adhesive tape and initiated by a red-hot needle. Strong deflagration or detonation of the compound usually indicates a valuable primary explosive. The safe and straightforward hot plate test only shows the behavior of the unconfined sample toward fast heating on a copper plate. It does not necessarily allow any conclusions on a compound‘s capability as a suitable primary explosive. The laser initiation experiments were performed with a 45 W InGaAs laser diode operating in the single-pulsed mode. The diode is attached to an optical fiber with a core diameter of 400 μm and a cladding diameter of 480 μm. The optical fiber is connected via a SMA type connecter directly to the laser and to a collimator. This collimator is coupled to an optical lens, which was positioned in its focal distance (f = 29.9 mm) to the sample. The lens is shielded from the explosive by a sapphire glass. Approximately 15 mg of the carefully pestled compound to be investigated was filled into a transparent plastic cap (PC), pressed with a pressure force of 1 kN and sealed by a UV-curing adhesive. The confined samples were irradiated at a wavelength of 915 nm, a voltage of 4 V, a current of 10–15 A and pulse lengths of 1–15 ms. The combined currents and pulse lengths result in an energy output of 4.5–126 mJ.

The obtained coordination compounds were washed with cold ethanol when stated, dried overnight in air and used for analytics without further purification.

CAUTION! *All investigated compounds are potentially explosive energetic materials, which show partly increased sensitivities toward various stimuli (e.g. elevated temperatures, impact, friction or electrostatic discharge). Therefore, proper security precautions (safety glass, face shield, earthed equipment and shoes, leather coat, Kevlar gloves, Kevlar sleeves and ear plugs) have to be applied while synthesizing and handling the described compounds.*

Dinitraminic acid (HDN, 1)

Amberlite IR120 hydrogen form (21 mL) was repeatedly suspended in water (50 mL) and decanted until the solution stayed colorless. The ion exchange material was transferred to a glass chromatography column with an estimated volume of 21 cm^3. The column was slowly loaded with ammonium dinitramide (2.98 g, 24.0 mmol) dissolved in water (25 mL). The ion exchange was carried out with a maximum drip rate of 2 to 3 drops per second. The eluate was rinsed with water until a neutral pH was reached. Depending on the amount of water used, solutions of dinitraminic acid up to 5 wt% were obtained in this way.

^{14}N-NMR (29 MHz, D_2O-d_2, 25 °C): δ (ppm) = −8.75.

General procedure for the preparation of complexes 2–7:

Basic copper(II) carbonate (55.3 mg, 0.25 mmol) and an aqueous solution of HDN (2 mL) were stirred mechanically until a clear solution was obtained. Stoichiometric amounts of the ligand dissolved in 2 mL water were added under stirring. The reaction mixtures were

left to crystallize, the solids were filtered off, washed with cold ethanol (2 mL) and dried in air.

$[Cu(NH_3)_4](DN)_2$ (2)

Blue needles of coordination compound **2** were isolated within a day. Yield: 72.2 mg (0.21 mmol, 86%).

DTA (5 °C min^{-1}) onset: 66 °C (endothermic), 179 °C (exothermic); IR (ATR, cm^{-1}): $\tilde{\nu}$ = 3340 (s), 3274 (m), 1614 (w), 1499 (s), 1424 (s), 1324 (m), 1272 (m), 1256 (s), 1169 (s), 1009 (s), 963 (m), 820 (m), 760 (s), 733 (s), 683 (vs), 459 (w), 434 (m); UV-Vis spectrum: λ_{max} = 550 nm; EA ($H_{12}CuN_{10}O_8$): calcd: H 3.52, N 40.75%; found: H 3.52, N 40.46%; BAM drophammer: 2 J; BDIS: > 200 mJ, BAM friction tester: 50 N; ESD: 1080 mJ (at grain size 500–1000 µm).

$[Cu(ATRI)_3](DN)_2$ (3)

Compound **3** was obtained as a light blue precipitate immediately after addition of the ligand. Yield: 116 mg (0.22 mmol, 90%).

DTA (5 °C min^{-1}) onset: 203 °C (exothermic); IR (ATR, cm^{-1}): $\tilde{\nu}$ = 3333 (w), 3287 (w), 3227 (w), 3127 (m), 1625 (w), 1505 (s), 1434 (s), 1395 (m), 1336 (w), 1165 (vs), 1088 (m), 1005 (s), 985 (s), 884 (s), 823 (m), 758 (s), 726 (m), 689 (w), 619 (s), 441 (w), 426 (w), 415 (w); UV-Vis spectrum: λ_{max} = 646 nm; EA ($C_6H_{12}CuN_{18}O_8$): calcd: C 13.65, H 2.29, N

47.77%; found: C 13.68, H 2.23, N 47.03%; BAM drophammer: 8 J; BDIS: 20 mJ; BAM friction tester: 80 N; ESD: 14 mJ (at grain size < 100 μm).

$[Cu(H_2O)_2(BTRI)_2](DN)_2$ (4)

Compound **4** was isolated as a blue precipitate directly after addition of the ligand. Yield: 134 mg (0.23 mmol, 92%).

Single crystal growth was achieved by overlaying an aqueous solution (4 mL) of copper(II) carbonate dissolved in dinitraminic acid with an ethanolic solution (4 mL) of 4,4'-bi(1,2,4-triazole) (BTz), separated by a mixture (2 mL) of water/ethanol (50/50). After two days blue blocks suitable for X-ray determination were obtained.

DTA (5 °C min^{-1}) onset: 129 °C (endothermic), 195 °C (exothermic); IR (ATR, cm^{-1}): $\tilde{\nu}$ = 3552 (w), 3435 (w), 3133 (m), 3108 (m), 1618 (w), 1504 (s), 1495 (s), 1428 (m), 1336 (w), 1316 (w), 1300 (w), 1212 (w), 1199 (w), 1163 (vs), 1084 (s), 1030 (s), 1003 (s), 954 (m), 937 (m), 885 (m), 870 (m), 849 (w), 816 (w), 758 (m), 743 (m), 679 (w), 613 (vs), 511 (m), 501 (m), 445 (m), 423 (w); UV-Vis spectrum: λ_{max} = 624 nm; EA ($C_8H_{12}CuN_{18}O_{10}$): calcd: C 16.46, H 2.07, N 43.18%; found: C 16.54, H 1.86, N 42.93%; BAM drophammer: 7 J; BDIS: 83 mJ; BAM friction tester: 144 N; ESD: 181 mJ (at grain size < 100 μm).

[Cu(MTZ)$_6$](DN)$_2$ (5)

Deep blue needles of complex **5** could be obtained after one week. Yield: 131 mg (0.16 mmol, 67%).

DTA (5 °C min^{-1}) onset: 114 °C (endothermic), 159 °C (exothermic); IR (ATR, cm^{-1}): $\tilde{\nu}$ = 3138 (w), 3030 (vw), 2970 (vw), 1509 (s), 1472 (m), 1431 (s), 1333 (w), 1306 (w), 1172 (vs), 1106 (vs), 1067 (w), 998 (s), 889 (m), 829 (m), 757 (s), 720 (m), 685 (s), 655 (vs), 469 (vw); UV-Vis spectrum: λ_{max} = 674 nm; EA ($C_{12}H_{24}CuN_{30}O_8$): calcd: C 18.48, H 3.10, N 53.87%; found: C 18.28, H 2.91, N 52.57%; BAM drophammer: 8 J; BDIS: > 200 mJ; BAM friction tester: 120 N; ESD: 203 mJ (at grain size 500–1000 µm).

[Cu(AMT)$_4$(H$_2$O)](DN)$_2$ (6)

Compound **6** was obtained as blue needles within a week, suitable for X-ray diffraction. Yield: 241 mg (0.17 mmol, 70%).

DTA (5 °C min^{-1}) onset: 89 °C (endothermic followed by exothermic); IR (ATR, cm^{-1}): $\tilde{\nu}$ = 3292 (w), 3244 (w), 2946 (vw), 1639 (m), 1512 (s), 1428 (m), 1378 (m), 1337 (m), 1322 (m), 1283 (m), 1173 (vs), 1126 (m), 1086 (w), 1001 (s), 924 (m), 829 (m), 759 (m), 741 (m), 717 (m), 676 (s), 585 (w), 479 (m); UV-Vis spectrum: λ_{max} = 643 nm; EA ($C_8H_{22}CuN_{26}O_9$): calcd: C 13.93, H 3.21, N 52.78%; found: C 14.30, H 2.94, N 52.67%; BAM drophammer: 2 J; BDIS: 28 mJ; BAM friction tester: 3 N; ESD: 250 mJ (at grain size < 100 µm).

[Cu(DN)$_2$(MAT)$_4$] • 2 H_2O (7)

Compound **7** crystalized in the form of blue-violet blocks within one week, suitable for X-ray determination. Yield: 257 mg (0.18 mmol, 73%).

DTA (5 °C min^{-1}) onset: 65 °C (endothermic), 114 °C (exothermic); IR (ATR, cm^{-1}): $\tilde{\nu}$ = 3566 (w), 3555 (w), 3531 (w), 3484 (w), 3443 (w), 3407 (m), 3380 (m), 3325 (m), 3271 (w), 3245 (m), 3238 (m), 3195 (w), 1634 (s), 1561 (m), 1539 (s), 1509 (s), 1440 (s), 1420 (s), 1383 (m), 1348 (w), 1327 (w), 1170 (vs), 1135 (s), 1075 (w), 1015 (s), 949 (m), 811 (m), 762 (m), 747 (m), 723 (w), 648 (m), 619 (w), 607 (w), 585 (w), 567 (w), 491 (m), 471 (w), 458 (m), 452 (m), 444 (m), 434 (m), 425 (w), 415 (w); EA ($C_8H_{24}CuN_{26}O_{10}$): calcd: C 13.57, H 3.42, N 51.44%; found: C 13.45, H 3.20, N 49.86%; BAM drophammer: 4 J; BDIS: > 200 mJ; BAM friction tester: 80 N; ESD: > 1500 mJ (at grain size 500–1000 µm).

[Cu(AMT)$_4$(H_2O)](DN)$_2$ (8)

Compound **8** was obtained as blue needles within a week, suitable for X-ray diffraction. Yield: 241 mg (0.17 mmol, 70%).

DTA (5 °C min^{-1}) onset: 89 °C (endothermic followed by exothermic); IR (ATR, cm^{-1}): $\tilde{\nu}$ = 3292 (w), 3244 (w), 2946 (vw), 1639 (m), 1512 (s), 1428 (m), 1378 (m), 1337 (m), 1322 (m), 1283 (m), 1173 (vs), 1126 (m), 1086 (w), 1001 (s), 924 (m), 829 (m), 759 (m), 741 (m), 717 (m), 676 (s), 585 (w), 479 (m); UV-Vis spectrum: λ_{max} = 643 nm; EA ($C_8H_{22}CuN_{26}O_9$): calcd: C 13.93, H 3.21, N 52.78%; found: C 14.30, H 2.94, N 52.67%; BAM drophammer: 2 J; BDIS: 28 mJ; BAM friction tester: 3 N; ESD: 250 mJ (at grain size < 100 µm).

[Cu(H$_2$O)$_2$(1,1-dtm)$_2$](DN)$_2$ (9)

Coordination compound **9** crystallized within three weeks in the form of blue blocks, suitable for X-ray determination. Yield: 87 mg (0.21 mmol, 85%).

DTA (5 °C min^{-1}) onset: 97 °C (endothermic), 165 °C (exothermic); IR (ATR, cm^{-1}): $\tilde{\nu}$ = 3441 (m), 3264 (w), 3108 (m), 3041 (w), 2991 (m), 1774 (vw), 1645 (m), 1536 (s), 1515 (s), 1490 (m), 1439 (s), 1424 (m), 1388 (w), 1375 (w), 1329 (w), 1304 (vw), 1239 (w), 1197 (s), 1185 (s), 1173 (vs), 1141 (s), 1104 (s), 1088 (s), 1036 (s), 1025 (s), 997 (s), 961 (m), 948 (m), 890 (s), 820 (m), 783 (s), 762 (m), 729 (s), 718 (s), 713 (s), 649 (m), 521 (m), 514 (m), 508 (m), 461 (m); UV-Vis spectrum: λ_{max} = 646 nm; EA ($C_6H_{12}CuN_{22}O_{10}$): calcd: C 11.70, H 1.96, N 50.04%; found: C 11.56, H 1.89, N 49.88%; BAM drophammer: 2 J; BDIS: 41 mJ; BAM friction tester: 40 N; ESD: > 1500 mJ (at grain size > 1000 µm).

[Cu(1,2-dtm)$_3$](DN)$_2$ (10)

Blue block of complex **10**, suitable for single crystal diffraction were obtained within one week. Yield: 157 mg (0.22 mmol, 86%).

DTA (5 °C min^{-1}) onset: 147 °C (exothermic); IR (ATR, cm^{-1}): $\tilde{\nu}$ = 3080 (w), 3066 (w), 3046 (w), 3026 (w), 2991 (w), 2977 (w), 1533 (m), 1515 (s), 1505 (s), 1475 (w), 1455 (m), 1450 (m), 1436 (m), 1425 (m), 1416 (m), 1378 (m), 1371 (m), 1353 (m), 1330 (w), 1313 (w), 1295 (w), 1286 (m), 1253 (w), 1203 (s), 1185 (s), 1169 (vs), 1160 (vs), 1128 (s), 1092 (s), 1052 (w), 1038 (m), 1003 (vs), 951 (m), 914 (m), 890 (m), 826 (m), 789 (s), 759 (m), 750 (s), 740 (s), 723 (m), 714 (m), 704 (m), 672 (s), 649 (m), 468 (vw), 458 (vw), 442 (w),
422 (vw), 403 (vw); UV-Vis spectrum: λ_{max} = 643 nm; EA ($C_9H_{12}CuN_{30}O_8$): calcd: C 14.77,

H 1.65, N 57.41%; found: C 14.53, H 1.66, N 57.22%; BAM drophammer: 2 J; BDIS: 28 mJ; BAM friction tester: 15 N; ESD: 270 mJ (at grain size 500–1000 μm).

[Cu(1,1-dte)$_3$](DN)$_2$ (11)

Compound **11** was obtained as blue blocks within five hours, suitable for X-ray determination. Yield: 116 mg (0.22 mmol, 90%).

DTA (5 °C min^{-1}) onset: 168 °C (exothermic); IR (ATR, cm^{-1}): $\tilde{\nu}$ = 3137(w), 3028(w), 2989(vw), 1515(s), 1496(m), 1436(s), 1368(w), 1319(w), 1288(w), 1269(w), 1175(vs), 1145(s), 1097(s), 1051(w), 993(s), 975(s), 937(m), 894(m), 825(w), 757(m), 720(m), 697(w), 683(m), 683(m), 672(m), 658(s), 637(m), 492(w); UV-Vis spectrum: λ_{max} = 643 nm; EA ($C_{12}H_{18}CuN_{30}O_8$): calcd: C 18.62, H 2.34, N 54.29%; found: C 19.09, H 2.27, N 54.74%; BAM drophammer: 2 J; BDIS: 28 mJ; BAM friction tester: 72 N; ESD: 76 mJ (at grain size < 100 μm).

[Cu(2,2-dte)$_3$](DN)$_2$ (12)

Compound **12** crystalized as blue block suitable for X-ray analysis within 12 h. Yield: 116 mg (0.22 mmol, 90%).

DTA (5 °C min^{-1}) onset: 150 °C (exothermic); IR (ATR, cm^{-1}): $\tilde{\nu}$ = 3023 (w), 2976 (w), 1514 (m), 1505 (m), 1479 (w), 1471 (w), 1464 (w), 1442 (m), 1428 (s), 1404 (m), 1376 (m), 1332 (m), 1309 (m), 1298 (m), 1278 (w), 1226 (w), 1172 (vs), 1145 (vs), 1055 (m), 1047 (m), 1003 (s), 974 (s), 934 (m), 901 (m), 824 (m), 757 (s), 726 (m), 705 (m), 690 (m),

677 (m), 655 (s), 550 (s), 517 (w), 505 (w), 485 (w); UV-Vis spectrum: λ_{max} = 643 nm; EA ($C_{12}H_{18}CuN_{30}O_8$): calcd: C 18.62, H 2.34, N 54.29%; found: C 18.75, H 2.39, N 54.15%; BAM drophammer: 2 J; BDIS: 25 mJ; BAM friction tester: 30 N; ESD: 250 mJ (at grain size 500–1000 µm).

[Cu(H_2O)$_2$(idtp)$_2$](DN)$_2$ (13)

Coordination compound **13** crystallized within two days in the form of blue rods, suitable for X-ray determination. Yield: 87 mg (0.13 mmol, 55%).

DTA (5 °C min^{-1}) onset: 113 °C (endothermic), 152 °C (exothermic); IR (ATR, cm^{-1}): $\tilde{\nu}$ = 3613 (w), 3449 (w), 3165 (w), 3118 (w), 3017 (w), 2994 (vw), 1785 (vw), 1639 (w), 1626 (w), 1512 (s), 1490 (s), 1463 (m), 1433 (s), 1394 (w), 1379 (w), 1361 (w), 1354 (w), 1325 (w), 1313 (w), 1284 (vw), 1171 (vs), 1165 (vs), 1131 (m), 1108 (s), 1095 (s), 1086 (s), 1043 (m), 1007 (s), 923 (m), 894 (m), 821 (m), 757 (m), 730 (w), 715 (w), 678 (m), 655 (m), 636 (m), 561 (vw), 505 (vw), 474 (w), 444 (w), 439 (w), 431 (w), 404 (vw); UV-Vis spectrum: λ_{max} = 646 nm; EA ($C_{10}H_{20}CuN_{22}O_{10}$): calcd: C 17.87, H 3.00, N 45.86%; found: C 18.06, H 2.83, N 45.94%; BAM drophammer: 2 J; BDIS: 180 mJ; BAM friction tester: 120 N; ESD: 317 mJ (at grain size < 100 µm).

[Cu(DN)$_2$(1,2-dtp)$_2$] (14)

Complex **14** was isolated after x days in the form of blue blocks. Yield: 280 mg (0.17 mmol, 69%).

DTA (5 °C min^{-1}) onset: 141 °C (exothermic); IR (ATR, cm^{-1}): $\tilde{\nu}$ = 3027 (w), 2991 (vw), 1524 (s), 1508 (s), 1504 (s), 1451 (s), 1438 (s), 1426 (s), 1378 (w), 1364 (w), 1355 (w), 1351 (w), 1342 (w), 1329 (m), 1307 (m), 1294 (w), 1238 (w), 1185 (vs), 1168 (vs), 1152 (vs), 1095 (s), 1055 (m), 1036 (w), 1026 (w), 999 (s), 990 (s), 979 (vs), 906 (s), 885 (m), 870 (m), 825 (s), 761 (s), 731 (s), 694 (s), 685 (m), 657 (s), 631 (s), 582 (w), 533 (w), 484 (w), 475 (w), 462 (w), 432 (m); UV-Vis spectrum: λ_{max} = 646 nm; EA ($C_{15}H_{24}CuN_{30}O_8$): calcd: C 22.08, H 2.96, N 45.86%; found: C 18.06, H 2.83, N 51.49%; BAM drophammer: 2 J; BDIS: > 200 mJ; BAM friction tester: 60 N; ESD: 840 mJ (at grain size 100–500 µm).

[Cu(dtb)$_3$](DN)$_2$ (15)

Complex **15** crystalized in the form of blue block suitable for X-ray diffraction after 12 hours. Yield: 178 mg (0.21 mmol, 83%).

DTA (5 °C min^{-1}) onset: 97 °C (endothermic), 147 °C (exothermic); IR (ATR, cm^{-1}): $\tilde{\nu}$ = 2960 (w), 2935 (vw), 1511 (m), 1505 (s), 1487 (m), 1471 (m), 1456 (m), 1444 (s), 1435 (m), 1422 (s), 1418 (s), 1377 (m), 1335 (m), 1324 (w), 1304 (w), 1266 (w), 1229 (w), 1174 (vs), 1162 (vs), 1134 (s), 1094 (vs), 1009 (s), 977 (s), 929 (m), 918 (m), 892 (m), 875 (s), 826 (m), 794 (m), 770 (m), 756 (s), 742 (m), 732 (m), 713 (s), 668 (m), 657 (s), 645 (m), 465 (m); UV-Vis spectrum: λ_{max} = 646 nm; EA ($C_{18}H_{30}CuN_{30}O_8$): calcd: C 25.19, H 3.52,

N 48.96%; found: C 25.13, H 3.45, N 49.15%; BAM drophammer: 2 J; BDIS: > 200 mJ; BAM friction tester: 240 N; ESD: > 1500 mJ (at grain size < 100 μm).

10. References

[S1] CrysAlisPRO (Version 171.33.41), Oxford Diffraction Ltd., **2009**.

[S2] A. Altomare, G. Cascarano, C. Giacovazzo, A. Guagliardi, *J. Appl. Crystallogr.* **1993**, *26*, 343–350.

[S3] (a) A. Altomare, G. Cascarano, C. Giacovazzo, A. Guagliardi, A. G. G. Moliterni, M. C. Burla, G. Polidori, M. Camalli, R. Spagna, *SIR97*, **1997**. (b) A. Altomare, M. C. Burla, M. Camalli, G. L. Cascarano, C. Giacovazzo, A. Guagliardi, A. G. G. Moliterni, G. Polidori, R. Spagna, *J. Appl. Crystallogr.* **1999**, *32*, 115–119.

[S4] (a) G. M. Sheldrick, SHELXL-97, University of Göttingen, Germany, **1997**. (b) G. M. Sheldrick, *Acta Cryst. A* **2008**, *64*, 112–122.

[S5] G. M. Sheldrick, *Acta Cryst. A* **2015**, *71*, 3–8.

[S6] A. L. Spek, PLATON, Utrecht University, The Netherlands, **1999**.

[S7] L. J. Farrugia, L. *J. Appl. Cryst.* **2012**, *45*, 849–854.

[S8] O. V. Dolomanov, L. J. Bourhis, R. J. Gildea, J. A. K. Howard, H. Puschmann, *J. Appl. Cryst*. **2009**, *42*, 339–341.

[S9] Empirical absorption correction using spherical harmonics, implemented in SCALE3 ABSPACK scaling algorithm (CrysAlisPro Oxford Diffraction Ltd., Version 171.33.41, **2009**).

[S10] APEX3, Bruker AXS Inc., Madison, Wisconsin, USA

[S11] NATO standardization agreement (STANAG) on explosives, impact sensitivity tests, no. 4489, 1st ed., Sept. 17, **1999**.

[S12] WIWEB-Standardarbeitsanweisung 4-5.1.02, Ermittlung der Explosionsgefährlichkeit, hier der Schlagempfindlichkeit mit dem Fallhammer, Nov. 8, **2002**.

[S13] OZM, http://www.ozm.cz, accessed Feruary **2021**.

[S14] BAM, http://www.bam.de, accessed February **2021**.

[S15] Military Standard 1751A (MIL-STD-1751A): safety and performance tests for qualification of explosives (high explosives, propellants and pyrotechnics), method 1016, Dec. 11, **2001**.

[S16] NATO standardization agreement (STANAG) on explosive, friction sensitivity tests, no. 4487, 1st ed., Aug. 22, **2002**.

[S17] WIWEB-Standardarbeitsanweisung 4-5.1.03, Ermittlung der Explosionsgefährlichkeit oder der Reibeempfindlichkeit mit dem Reibeapparat, Nov. 8, **2002**.

[S18] UN Model Regulation: Recommendations on the Transport of Dangerous Goods – Manual of Tests and Criteria, section 13.4.2.3.3, **2015**.

[S19] Impact: insensitive > 40 J, less sensitive ≥ 35 J, sensitive ≥ 4 J, very sensitive ≤ 3 J; Friction: insensitive > 360 N, less sensitive = 360 N, sensitive < 360 N and > 80 N, very sensitive ≤ 80 N, extremely sensitive ≤ 10 N. According to the UN Recommendations on the Transport of Dangerous Goods, 5th ed., **2009**.
